# New Proposal of Epiphytic Bromeliaceae Functional Groups to Include Nebulophytes and Shallow Tanks

**DOI:** 10.3390/plants11223151

**Published:** 2022-11-17

**Authors:** Casandra Reyes-García, Narcy Anai Pereira-Zaldívar, Celene Espadas-Manrique, Manuela Tamayo-Chim, Nahlleli Chilpa-Galván, Manuel Jesús Cach-Pérez, Marypaz Ramírez-Medina, Ana Maria Benavides, Peter Hietz, Gerhard Zotz, José Luis Andrade, Catherine Cardelús, Rodolfo de Paula Oliveira, Helena J. R. Einzmann, Valeria Guzmán Jacob, Thorsten Krömer, Juan P. Pinzón, Juliano Sarmento Cabral, Wolfgang Wanek, Carrie Woods

**Affiliations:** 1 Unidad de Recursos Naturales, Centro de Investigación Científica de Yucatán, 43 St., Chuburna de Hidalgo, Mérida 97205, Mexico; 2 El Colegio de la Frontera Sur, Guineo, Second Section, Villahermosa 86280, Mexico; 3 Jardín Botánico de Medellín, 73 St., Medellín 50035, Colombia; 4 Department of Integrative Biology and Biodiversity Research, University of Natural Resources and Life Sciences, Gregor-Mendel-Straße 33, 1180 Vienna, Austria; 5 Functional Ecology Group, Institute of Biology and Environmental Sciences, University of Oldenburg, Box 2503, D-26111 Oldenburg, Germany; 6 Smithsonian Tropical Research Institute, Panama City 32402, Panama; 7 Departments of Biology and Environmental Studies, Colgate University, 13 Oak Dr E Ext, Hamilton, NY 13346, USA; 8 Departament of Botany, University of Brasilia, Brasilia 70910-900, Brazil; 9 Biodiversity, Macroecology and Biogeography, University of Goettingen, 37073 Göttingen, Germany; 10 Centro de Investigaciones Tropicales, Universidad Veracruzana, Veracruz 91090, Mexico; 11 Departamento de Botánica, Campus de Ciencias Biológicas y Agropecuarias, Universidad Autónoma de Yucatán, Road Mérida-Xmatkuil km 15.5, Mérida 97315, Mexico; 12 School of Biosciences, University of Birmingham, Birmingham B15 2TT, UK; 13 Center of Microbiology and Environmental Systems Science, University of Vienna, Djerassipl, 1030 Vienna, Austria; 14 Department of Biology, University of Puget Sound, 1500 N Warner St., Tacoma, WA 98416, USA

**Keywords:** bromeliads, *Tillandsia*, epiphytes, photosynthetic pathway, CAM, ecosystems, functional traits, fog

## Abstract

The Bromeliaceae family has been used as a model to study adaptive radiation due to its terrestrial, epilithic, and epiphytic habits with wide morpho-physiological variation. Functional groups described by Pittendrigh in 1948 have been an integral part of ecophysiological studies. In the current study, we revisited the functional groups of epiphytic bromeliads using a 204 species trait database sampled throughout the Americas. Our objective was to define epiphytic functional groups within bromeliads based on unsupervised classification, including species from the dry to the wet end of the Neotropics. We performed a hierarchical cluster analysis with 16 functional traits and a discriminant analysis, to test for the separation between these groups. Herbarium records were used to map species distributions and to analyze the climate and ecosystems inhabited. The clustering supported five groups, *C_3_ tank* and *CAM tank* bromeliads with deep tanks, while the atmospheric group (according to Pittendrigh) was divided into *nebulophytes*, bromeliads with *shallow tanks*, and bromeliads with *pseudobulbs*. The two former groups showed distinct traits related to resource (water) acquisition, such as fog (nebulophytes) and dew (shallow tanks). We discuss how the functional traits relate to the ecosystems inhabited and the relevance of acknowledging the new functional groups.

## 1. Introduction

Functional groups provide important insights into plant function, structure, and interaction with the surrounding environment and can be used to simplify complex communities and gain a better understanding of the underlying processes [1,2,3]. Shared morphological, anatomical, physiological, and ecological traits within groups of species also provide insights into evolutionary processes in response to environmental conditions, highlighting the interconnection of traits and their trade-offs [4,5,6]. Epiphytes are subject to specific selective pressures by growing on trees disconnected from forest soils. Because water and nutrients are only available in pulses, most vascular epiphytes are drought tolerant and grow slowly [7]. Discontinuous resource supply has also given rise to several traits for the collection and storage of water, coupled with water saving processes and specialized nutrient acquisition mechanisms [8,9]. Plant size may also be constrained by the fragility of the supporting branches [6,10,11,12].

The family Bromeliaceae represents a good model for the study of functional groups and adaptive radiation. This family is the second most numerous in epiphytic species [13,14] and has a wide array of vegetative forms that inhabit diverse habitats across the tropical and subtropical Americas [15,16]. As early as 1948, Pittendrigh described four functional groups of bromeliads and related these to different environments along a precipitation gradient (1000–6350 mm y^−1^) in Trinidad [17]. Two of the groups pertain to epiphytic species. The tank-absorbing trichome group (also known as type III) encompasses species that form a water-holding vessel (“tank”) between their overlapping leaf bases, which are arranged in a rosette. Water and nutrients are absorbed directly from the tank, via specialized absorbing leaf trichomes characteristic of the Bromeliaceae [18,19,20]. The tank provides a more permanent water source in between rain events. The second epiphytic group is the atmosphere-absorbing trichome group (also known as type IV), which lacks a tank, has highly reduced root systems, and has high trichome coverage that absorbs water and nutrients during precipitation events. These functional groups were formally revised by Benzing [21], who divided the tank-absorbing trichome species into two groups, depending on their photosynthetic pathway, i.e., the C_3_ tank and the crassulacean acid metabolism (CAM) tank group.

Physiological studies have validated the divergence between tank and atmospheric functional groups. Tank species have higher photosynthetic rates than coexisting atmospherics, while atmospherics maintain photosynthetic activity longer during extended drought periods, even when exhibiting low leaf water content [22,23,24,25,26,27]. The reduced plant size in atmospherics relates to neoteny and is concomitant with tighter mesophyll cell packing and vasculature reduction, which contributes to higher water-use efficiency (WUE), but also to lower photosynthetic capacity [9]. The higher water-use efficiency of CAM epiphytes is reflected in their distribution, where they are more abundant at the drier end of the precipitation gradient. In contrast, C_3_ species dominate mesic and humid sites [28,29].

Besides the photosynthetic pathway, diverging strategies are found within the tank and the atmospheric groups that may warrant further analysis of functional groups in epiphytic Bromeliaceae. Tank bromeliad species from the drier spectrum, i.e., from seasonally dry forests, were underrepresented in Pittendrigh’s study and often possess reduced tanks and show traits similar to the atmospherics, such as high trichome coverage throughout the whole leaf blade and succulence [24]. Small tanks promote dew condensation on the leaf surfaces more effectively than coexisting atmospherics, as their thinner leaves cool faster overnight, resulting in longer intervals under dew point temperature [27]. Dewfall is more reliable than rainfall in many seasonal forests [27,30,31] and does not require high tank water holding capacities. Large tanks may be less advantageous in forests where low daytime humidity contributes to tank water evaporation [29] and where thermoregulation of larger leaves is more difficult under high temperatures [32].

Some atmospheric species exhibit a narrow-leaf syndrome, which is defined by long, thin, narrow leaves that are displayed high in the canopy and effectively intercept small fog droplets by reducing the leaf boundary layer [33,34]. Species with this syndrome, also referred to as nebulophytes [35], are found in different plant families (e.g., Agavaceae and Arecaceae [34]), and in terrestrial as well as epiphytic species, but are well represented among atmospheric Bromeliaceae. Thin leaves in the nebulophytes increase leaf mobility under wind currents and promote fog interception [34], thus contrasting with the reliance of atmospherics on well-developed hydrenchyma. Nebulophytic bromeliads are mostly found in the genus *Tillandsia* and are dominant in desert areas where fog constitutes the major water input [36,37,38]. Nebulophytes are also well represented in forest ecosystems with fog formation [24].

Pseudobulbous bromeliads are recognized as a distinct morphology within the atmospheric species [39,40]. The pseudobulbs are formed by involute leaves, displayed in a rosette, which generate ant-housing cavities, forming a facultative symbiosis where the bromeliad benefits from the nutrient inputs by the ants (e.g., *T. butzii* and *T. caput-medusae*; [39]).

Additionally, an alternate classification system was proposed for the genus *Tillandsia* based on trichome density and leaf area [41]. This classification recognized five groups, the first two having lower trichome coverage, corresponding with tank species, while the other three were atmospherics with increasingly high trichome coverage. Yet, to extrapolate this classification to other genera in the Bromeliaceae family would call for further research, due to the very different trichome properties among the genera, some being hygroscopic and others hydrophobic [42,43]. 

Both Pittendrigh [17] and Benzing [21] recognized the existence of subtypes within the main functional groups they proposed, such as “ephemeral tanks”, “atmospheric and tank intermediates”, and “dew- or rain-type atmospherics”, although these subtypes were not considered differentiated enough to constitute separate functional groups. One of the main advantages of using functional groups is the capacity to reduce inherent species variation by grouping them into larger categories that describe most of the variation, while not considering smaller deviations. However, refining the existing functional groups is relevant, if the divergent syndromes show anatomical, physiological, and ecological distinctness and are widely represented in the family.

Multivariate analyses of functional traits may provide a method to recognize whether proposed new groups have divergent syndromes from the previously postulated groups. Recent studies have used vascular epiphyte functional traits to compare them to other life forms such as herbs and trees [6], to compare epiphyte traits across environments [44,45,46,47], in relation to hosts [48] and relative to vertical gradients within the canopy [47]. Because most epiphytes are non-woody and usually show reduced stem and root systems, these studies have mostly centered on leaf traits. Agudelo [44] constructed functional groups using a large set of epiphytes from different families; the groups segregated following the main strategies, with rapid to slow resource acquisition as described by the leaf economics spectrum (LES; [49]). C_3_ and CAM photosynthetic pathways were also found to capture much of the interspecies variation observed in other traits [45]. Nevertheless, traits that are central to the Bromeliaceae functional groups have not been considered in these multivariate analyses, as they lack relevance in other families, i.e., tank water holding capacity and trichome density.

In the current study, we used a global database of epiphytic traits to reevaluate the classification of functional groups in the Bromeliaceae family. Our hypotheses were that: (1) traits of the nebulophytic species will separate them from the more succulent pseudobulbous atmospherics, due to their narrow, long, thin leaves, which reflect their dependance on fog, rather than rain; (2) shallow tank species mainly acquire dew, enabled by small tanks and thin leaves that cool quickly. These traits will differentiate them from the other two atmospheric groups; (3) tank species will segregate in CAM and C_3_ species. To test these hypotheses, we performed an unsupervised hierarchical cluster analysis to define functional groups based on functional traits such as tank capacity, leaf traits (area, thickness, shape), trichome and stomata density, leaf nutrient content (N, P, C), and δ^13^C and δ^15^N signatures as further physiological proxies for photosynthetic type and nitrogen nutrition. We also analyzed the climatic distribution and ecosystem prevalence across the obtained functional groups.

## 2. Results

### 2.1. Functional Groups and Associated Functional Traits

We obtained 25 functional traits that are related to photosynthesis, water use, plant size, and water storage (Table 1, 16574 single trait observations). The data belonged to 204 species and 23 genera of epiphytic Bromeliaceae, representing measurements taken from the whole geographic range of the family, from North to South America. The most diverse genus *Tillandsia* represented 57% of the records, followed by *Guzmania* (13%), *Aechmea* (9%), *Catopsis* (7%), and *Racinaea* (6%, Table 2). The data also represented the range of trait variation found within the family, from species with a height of 0.03–2.7 m in adult plants, with CAM and C_3_ representatives, found in contrasting environments such as tropical deserts to temperate montane forests.

**Figure 1:**
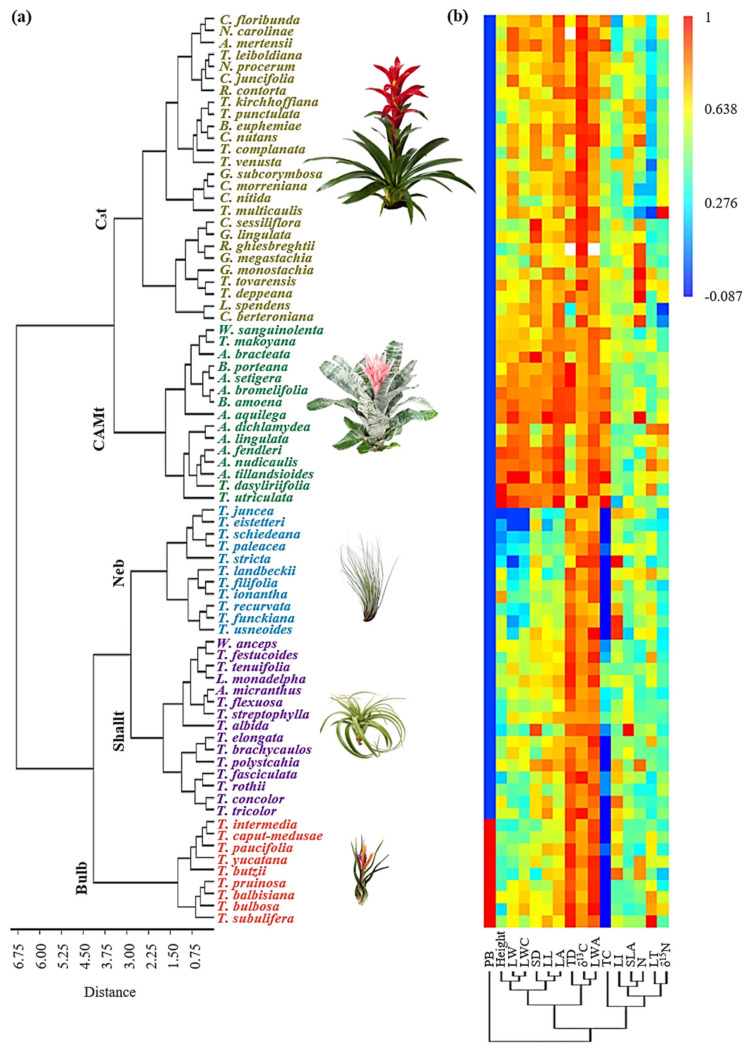
Hierarchical cluster analysis with Ward’s algorithm of major traits that separated 76 epiphytic Bromeliaceae species into five functional groups. (**a**) Species clustering; (**b**) trait clustering and heatmap of species vs. functional traits. Neb = nebulophytes; Bulb = pseudobulbs; ShallT = shallow tanks; C3T = C_3_ tanks, and CAMT = CAM tanks. For trait abbreviations, see Table 1.

The hierarchical cluster analysis separated the 76 species considered (subset used for this analysis) into deep tank (C_3_ and CAM tanks) and atmospherics (mostly CAM; Figure 1a). This first separation was related to a cluster of higher values in adult plant height, leaf area, leaf width, and stomata density in the deep tank species, compared to the atmospherics (Figure 1b). Leaf trichome density, leaf water content per area (LWA), and δ^13^C represented another cluster of traits, which contributed to the separation of C_3_ (mostly *Guzmania*) and CAM (mostly *Aechmea*) deep tanks, as C_3_ plants had higher δ^13^C (absolute values of δ^13^C were used here, so these represent more negative δ^13^C values), but lower succulence and trichome density. A third cluster of traits associated tank capacity with specific leaf area, leaf N, δ^15^N, and leaf thickness. Atmospherics were divided into 1) nebulophytes (e.g., *Tillandsia juncea* and *T. recurvata*) with narrow leaves and small leaf area, 2) pseudobulbous bromeliads (e.g., *Tillandsia paucifolia* and *T. balbisiana*) that have involute leaf bases, and 3) shallow tanks (e.g., *Tillandsia fasciculata* and *T. polystachia*) that hold a small volume of water between their leaf bases. Shallow tanks were mostly CAM, with their tank capacity varying from 2 to 61 mL, though two C_3_ species also fell into this category: *Wallisia anceps* and *Lemeltonia monadelpha* showed very little tank capacity (2 mL), and exhibited a high leaf index (LI = leaf length/leaf maximal width) and low δ^15^N compared to the C_3_ (deep) tank group. However, most C_3_ species with low to intermediate tank capacity (between 6 and 60 mL) were grouped within C_3_ (deep) tank species. Thus, the threshold in tank capacity for the shallow tank functional group is 2–61 mL for CAM species and includes C_3_ species with negligible tank capacity. 

Three deep tank species with CAM were classified in the C_3_ tank group (*Aechmea mertensi*, *Nidularium procerum*, and *Neogerelia carolinae*), and in this study were reclassified to match their photosynthetic pathway (CAM deep tank group), to follow a more intuitive method of classification. Additionally, *Tillandsia festucoides* was reclassified as a nebulophyte (being placed in the shallow tank group), due to the morphological similarity to other nebulophytic species (high leaf index, acicular leaves). Even with these reclassifications, the discriminant analysis (DA) confirmed the separation of the five groups (Table S1). The squared Mahalanobis distances test confirmed that the shallow tank species were more similar to the other atmospherics, with Mahalobis distances of 12 and 18 to pseudobulbs and nebulophytes, respectively, compared to 30 and 32 to the C_3_ and CAM deep tanks, respectively.

We classified the remaining 128 species into the five groups obtained based on the following most influential traits: 1) tank capacity, 2) photosynthetic pathway, 3) leaf bases forming a pseudobulb, and 4) presence of narrow, acicular leaves (see Table 3). Of the 204 species with functional traits, 38% were C_3_ tanks, 29% CAM tanks, 5% were pseudobulbs, 11% nebulophytes, and 16% shallow tanks (Table S2). Deep CAM tanks were most frequently found in species of the genus *Aechmea* (48%, 29 species) and large C_3_ tanks in *Guzmania* (40%, 31 species). All of the pseudobulbs, 95% of the nebulophytes, and 51% of the shallow tanks were attributed to species of *Tillandsia*, the genus with the most species in our database.

**Figure 2:**
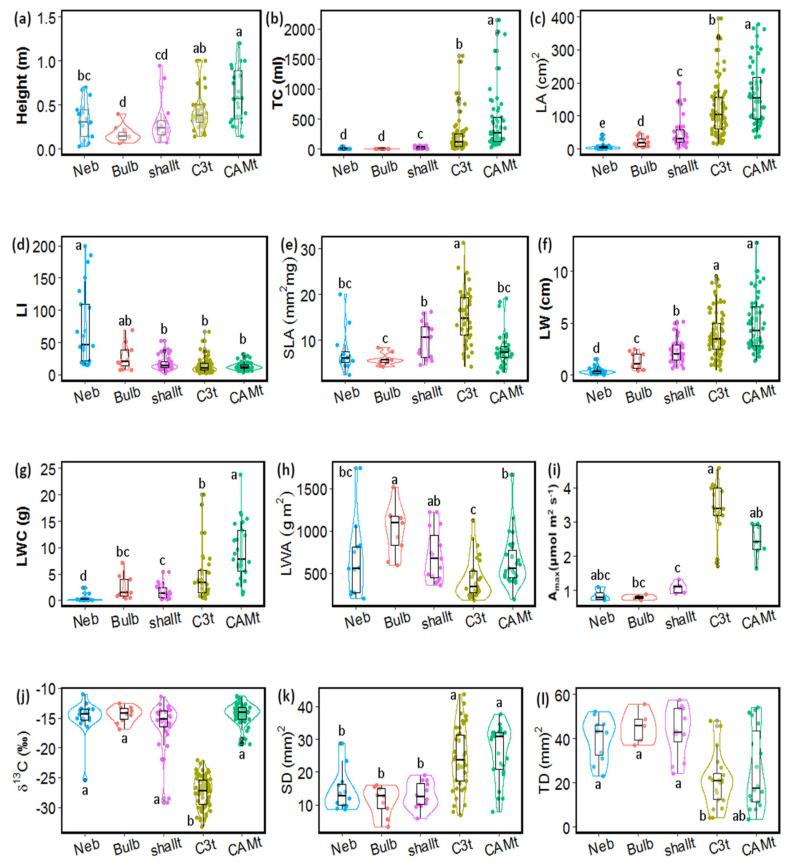
Comparison of functional traits between five functional groups of epiphytic Bromeliaceae, showing mean values for each species. (**a**) Height = adult plant height; (**b**) TC = tank capacity; (**c**) LA = leaf area; (**d**) LI = leaf index; (**e**) SLA = specific leaf area; (**f**) LW = leaf width; (**g**) LWC = total leaf water content; (**h**) LWA = leaf water content on area basis; (**i**) A_max_ = light saturated photosynthetic rate per leaf area; (**j**) δ^13^C = leaf carbon isotope signature; (**k**) SD = stomatal density; and (**l**) TD = leaf trichome density per functional group. Neb = nebulophytes; Bulb = pseudobulbs; ShallT = shallow tanks; C3T = C_3_ tanks and CAMT = CAM tanks. Groups with different letters are significantly different (Wilcoxon and Tukey HSD post-hoc tests, *p* < 0.05). Extreme data points were not depicted in the graph to help the visualization of the data for: LA, LWC, SD, and SLA.

Sixteen of the 23 continuous functional traits (binary and categorical traits not included here) were significantly different among the functional groups (Figure 2 and Figure S1, data from 204 species). CAM and C_3_ (deep) tank species had higher adult plant height, leaf width, stomatal density, and light saturated photosynthetic rate per leaf area (A_max_), compared with all other groups (Figure 2). CAM tanks had higher tank capacity, total leaf water content (LWC), leaf area, and leaf length than all other groups, while C_3_ tanks had intermediate tank capacity, the lowest trichome density, δ^13^C and leaf thickness, and the highest specific leaf area (SLA) of all the groups. Shallow tanks had lower tank capacity than the deep tank groups, and intermediate values of LWC, A_max_, leaf area, and width, compared with deep tanks and other atmospherics. Both nebulophytes and pseudobulbs lacked tank capacity, but nebulophytes had a higher leaf index and lower leaf area, leaf width, and leaf water content per area or total, compared to the pseudobulbs. Leaf carbon content (expressed in % of leaf dry matter) was higher in C_3_ tanks compared to nebulophytes and shallow tanks, while δ^15^N was significantly lower in nebulophytes, compared to C_3_ tanks.

No differences were found in leaf nutrient N (Kruskal–Wallis, H = 7.3, *p* > 0.05), *p* (Kruskal–Wallis, H = 1.3, *p* > 0.05) or chlorophyll content (Kruskal–Wallis, H = 23.8, *p* > 0.05) among the functional groups (Figure S1). Leaf force to punch, leaf dry mass, and stomatal size (either width or length) were also not significantly different among the groups (*p* > 0.05).

### 2.2. Correlations between Functional Traits

**Figure 3:**
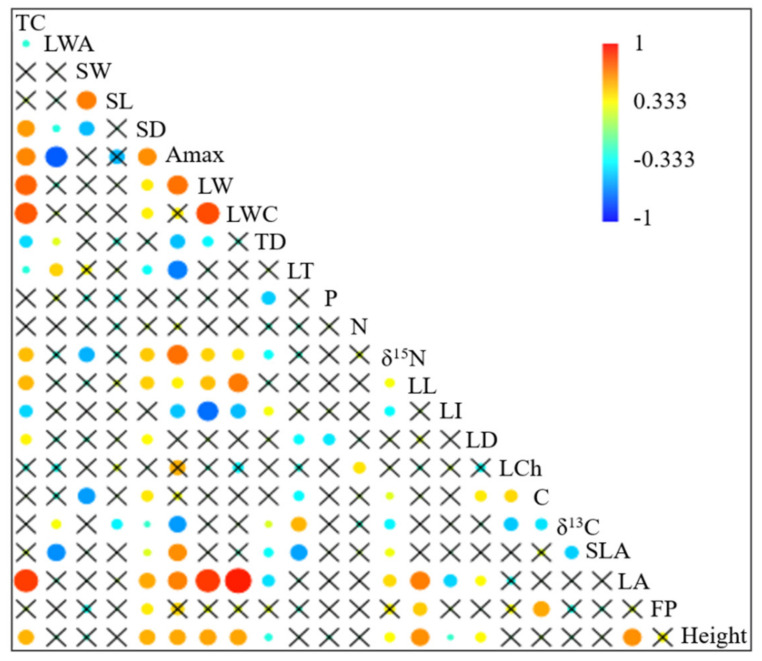
Spearman rank order correlations. Height = adult plant height; FP = force to punch; LA = leaf area; LD = leaf dry matter content; SLA = specific leaf area; δ^13^C = leaf carbon isotope signature; C = leaf carbon content per leaf dry mass, LCh = leaf chlorophyll content per leaf dry mass; LI = leaf index (leaf length/leaf width); LL = leaf length; δ^15^N = leaf nitrogen isotope signature; N = leaf nitrogen content per leaf dry mass; *p* = leaf phosphorus content per leaf dry mass; LT = leaf thickness, TD = leaf trichomes density, LWC = total leaf water content, LW = leaf width, A_max_ = light saturated photosynthetic rate per leaf area; SD = abaxial stomatal density; SL = stomatal length; SW = stomatal width; LWA = leaf water content on area basis; TC = tank capacity. Crossed out correlations have *p* > 0.05. *p* values and Spearman’s correlation coefficients are shown in Table S3.

**Figure 4:**
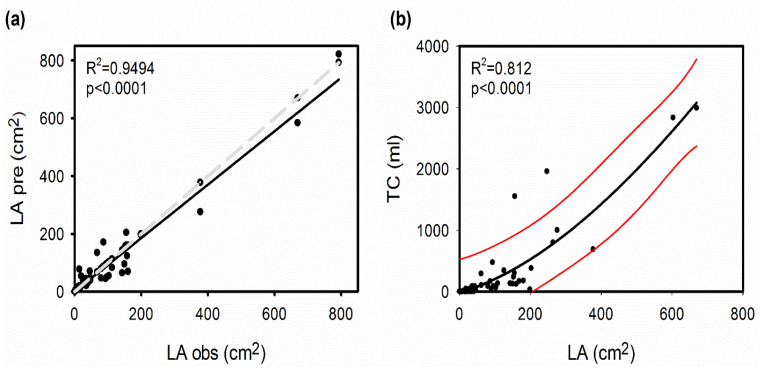
Regression models for tank capacity estimation. (**a**) Linear relationship between predicted leaf area (LA pre) and observed leaf area (LA obs). (**b**) Polynomial regression between tank water holding capacity (TC) and leaf area (LA). The broken line in (**a**) represents the 1:1 relationship between observed and predicted LA values, red lines in (**b**) represent 95% confidence intervals.

**Figure 5:**
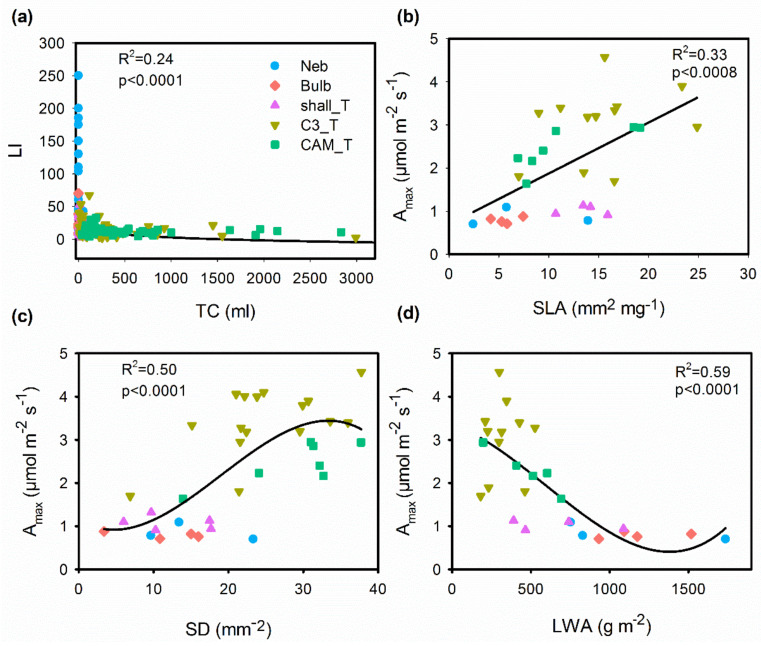
Significant relationships of functional trait across the five functional groups of epiphytic Bromeliaceae; (**a**) leaf index (LI) versus tank capacity (TC); (**b**) light saturated photosynthetic rate per leaf area (A_max_) versus specific leaf area (SLA); (**c**) A_max_ versus stomatal density (SD); (**d**) A_max_ versus leaf water content on area basis (LWA). Each point corresponds to the mean value per species. Point colors correspond to the following functional groups: Bulb = pseudobulbs (pink); C3_T = C_3_ tanks (yellow); Shall_T = shallow tanks (purple); CAM_T = CAM tanks (green); Neb = nebulophytes (blue).

Spearman rank correlations showed significant correlations in 87 out of 253 pairs of traits, yet strong correlations (*p* < 0.05, R^2^ > 0.60) were not as frequent and mostly included measures of plant size (Figure 3, Table S3). Tank capacity was positively correlated with leaf area (Figure 4), and leaf area was the best predictor of tank water holding capacity (R^2^ = 0.81). However, as expected, tank capacity also correlated positively with other traits related to leaf size (LW, LL), leaf dry matter content, and plant height (Figure 3). A trade-off was observed between tank capacity and leaf index (LI), where species with bigger tanks had very low leaf indices, and as leaf index increased tank capacity diminished (Figure 5a). Stomatal density and SLA were positively correlated with A_max_ (Figure 5b,c), and species with high LWA had lower A_max_ (Figure 5d). Isotopic values, considered as physiological proxies, were correlated with many traits. δ^13^C correlated negatively with leaf C (% dry mass), leaf chlorophyll concentration (LCh), SLA, δ^15^N, A_max_, stomatal density and length, and positively to trichome density, leaf thickness and LWA. δ^15^N correlated negatively with leaf index, trichome density, and stomatal width, and positively with tank capacity, A_max_, leaf width, stomatal density, SLA, LA, and adult plant height. In contrast, leaf N was only weakly positively correlated with leaf chlorophyll concentration.

### 2.3. Functional Groups’ Habitat and Distribution

**Figure 6:**
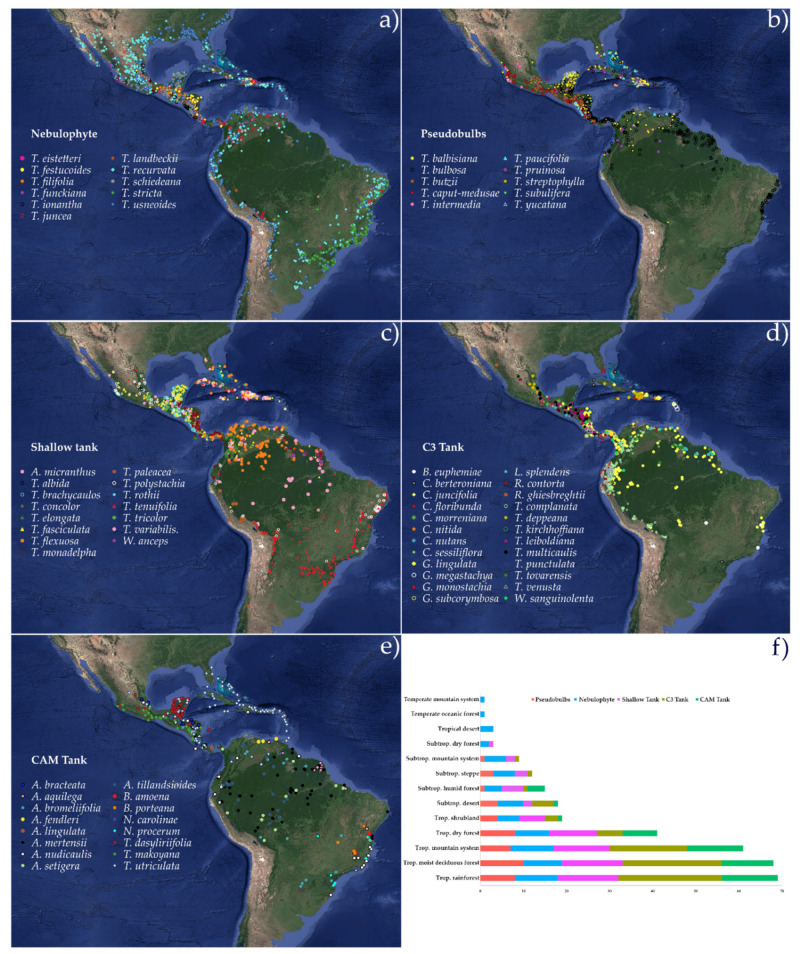
Geographical distribution of the functional groups: (**a**) nebulophytes; (**b**) pseudobulbs; (**c**) shallow tanks; (**d**) C_3_ tanks; (**e**) CAM tanks, and (**f**) species richness per functional groups in ecological zones [50].

**Figure 7:**
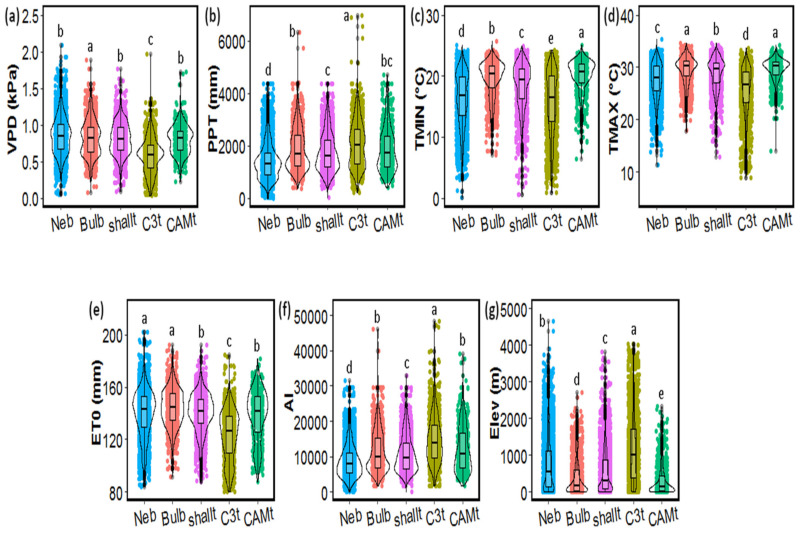
Comparison of environmental variables defining the average distribution area of the five functional groups of epiphytic Bromeliaceae, depicting environmental raw data. (**a**) VPD = vapor pressure deficit; (**b**) PPT = precipitation; (**c**) TMIN = minimum temperature; (**d**) TMAX = maximum temperature; (**e**) ET0 = evapotranspiration; (**f**) AI = aridity index; and (**g**) Elev = elevation per functional group. Neb = nebulophytes, Bulb = pseudobulbs, ShallT = shallow tank, C3T = C_3_ tanks, and CAMT = CAM tanks. Groups with different letters are significantly different (Wilcoxon post-hoc test, *p* < 0.05).

Herbarium and published records of the distribution of the 76 species first considered for cluster analysis yielded 8397 records of occurrence across the Americas. Records were more abundant in Mexico and Central America, while South America was underrepresented. Despite the possible bias from uneven specimen distribution, the large number of records and wide representation enabled preliminary, exploratory conclusions of differences in climatic and range distribution. The nebulophytic functional group included widely distributed species (Figure 6a), which were found in environments where other groups were largely excluded (Figure 6f). *Tillandsia usneoides* was the only species recorded in temperate environments (temperate montane systems and oceanic forests), and was located at higher latitudes than the rest of the species in the southern USA. *Tillandsia landbeckii* and *T. recurvata* were found in tropical (Atacama Desert, Chile) and subtropical (Chihuahuan desert, Mexico) deserts, respectively, colonizing the driest extreme of the ecosystem spectrum. Montane forests, with a frequent occurrence of fog, were also important ecosystems for nebulophytic species. Thus, nebulophytes were found at the sites with lowest precipitation and aridity index (AI), lower AI values indicating more arid environments, and highest vapor pressure deficit (VPD) of the groups (Figure 7). On the other end of the spectrum from the nebulophytes were the C_3_ tanks, which were highly related to montane and wet environments (Figure 6d,f), thus thriving under conditions of highest elevation, precipitation, and AI, and the lowest VPD, evapotranspiration, and minimum (T_min_) and maximum (T_max_) temperatures of all the functional groups (Figure 7).

CAM tanks and pseudobulbs were located in environments at low elevations and high temperatures (T_min_ and T_max_). Shallow tanks inhabited similar environments to CAM tanks and pseudobulbs, but at intermediate values of altitude, temperature, and AI. All of these groups were highly represented in tropical rainforests, in tropical moist deciduous forests, and in tropical montane forests (Figure 6f). CAM tanks were also frequent in tropical dry deciduous forests and became rare or absent in other temperate or drier ecosystems.

## 3. Discussion

The unsupervised hierarchical clustering approach supported previous classifications, where the largest variation in the functional traits of epiphytic Bromeliaceae is represented by the tank/atmospheric trade off. Tank species have larger sizes, bigger leaves which form bigger tanks, which provide a more stable water supply and relate to higher photosynthetic rates (Figure 1). In contrast, atmospherics show CAM photosynthesis, a reduction in size, stomatal density and photosynthetic rates, and a higher trichome density. Smaller clusters defined functional groups within these larger groups. In agreement with our hypotheses, atmospherics separated into shallow tanks, pseudobulboid species, and nebulophytes and deep tank species into C_3_ tank and CAM tank groups (Figure 1, Table 3). With these five groups, diverging functional strategies within the tank and atmospheric groups are represented in the trait space and the differences found in functional traits aligned with environmental differences in the species range distributions (Figure 6 and Figure 7).

Although we analyzed only a subset of species from this very diverse family, the trait differences clearly defined five functional groups. Given the large range of species’ sizes and shapes represented in this study, and the distribution along the entire subtropical and tropical range of the American continent, our data represent most of the variation within the family.

The nebulophytic functional group was characterized by a high leaf index (mean value of 83 for nebulophytes and 15 for non-nebulophytes), an important trait related to the narrow-leaf syndrome and fog interception [34]). However, some species that are well documented as fog-dependent, such as *Tillandsia recurvata* [38,51] and *Tillandsia landbeckii* [36,52], had modest LI values of 15–19, similar to those found in other groups. Leaf width, leaf area, and total leaf water content (LWC) were also characteristically low for all nebulophytic species. Thus, species in this group cannot rely on substantial water reserves, as small leaves will not allow a tank to form and also limit the amount of water that can be stored in tissues. Most nebulophytes belong to the genus *Tillandsia* and exhibit CAM photosynthesis (Table S2). Within the genus, there is evidence that the nebulophytic syndrome evolved repeatedly, as clusters of nebulophytic species are observed in the three subgenera *Tillandsia*, *Diaphoranthema*, and *Anoplophytum* [53,54]. The nebulophytic cluster in the subgenus *Tillandsia* includes a set of species that exhibit the highest leaf indices (e.g., *Tillandsia chaetophylla*, *T. eistetteri*, and *T. juncea*; all with LI > 100) and that are closely related [55]. These species are mainly found in tropical rainforests, and in montane, moist deciduous, and dry forests. In contrast, the nebulophytic species within the subgenus *Diaphoranthema* (*Tillandsia landbeckii*, *T. recurvata*, and *T. usneoides*) have also colonized subtropical and temperate montane and oceanic forest ecosystems, and can be abundant in deserts [35,36,52,56], ecosystems which are largely not colonized by most other epiphytic Bromeliaceae. *Diaphoranthema* species have lower LI than most nebulophytes, but are convergent in other traits such as reduced leaf area, leaf width, leaf water content, and δ^15^N, as they grouped together closely with other nebulophytes in the hierarchical cluster.

The evolutionary success of nebulophytic species is reflected in their geographic distribution (Figure 6), as this functional group is the most widespread geographically and across ecosystems. Large body plan changes are observed in *T. usneoides*, the leaves of this species not being arranged in a rosette, but along long sympodial stems that effectively form a meters-long biological fog-mesh. This species has a wide geographic distribution and was the only species in this study to inhabit sites where minimum temperatures fall below 0 °C. Frost has been recognized as a limiting factor for the distribution of most vascular epiphytes and particularly for Bromeliaceae in temperate climates [57,58]. In the distribution maps, *T. usneoides* has a larger high latitude range in North America compared to all other species (with the exception of a few records of *T. recurvata*, Figure 6). However, the mechanisms that enable moderate frost tolerance in this species remain unknown.

Species in the pseudobulbs functional group all belong to the genus *Tillandsia* and exhibit CAM photosynthesis and higher leaf thickness, leaf area, and LWA than nebulophytes. These differences highlight contrasting water acquisition and storage strategies among these non-tank forming atmospheric groups. Pseudobulbous species rely on internal water sources in the absence of rain [27], while nebulophytes generally have low degrees of succulence. Thus, even when nebulophytes are abundant in deserts, environmental data indicate that succulents are more resistant to high temperatures and evapotranspiration demands (Figure 7). In contrast, nebulophytes are generally limited to areas where high elevation and/or low minimum temperatures enable frequent fog/dew formation (Figure 7, [25,34]), and some species may rapidly desiccate under low relative humidity [59].

The main trait that delimited the pseudobulbs functional group in the cluster analysis was the presence of the pseudobulb. This may limit the inclusion of species that have similar water-use strategies but lack a pseudobulb, as this group had the least number of representatives (11 species) when we classified the 204 species. The pseudobulb is related to myrmecophily in some of the species [39,60], though expected increased nutrient inputs were not reflected in higher leaf N concentrations, compared to other atmospherics (Figure S1). However, species from other functional groups may also be associated with ant-gardens (e.g., *Tillandsia flexuosa* and some *Aechmea* species; [21,60]).

Previous classifications of the bromeliad functional groups included shallow tanks (tank-atmospheric intermediates) as atmospheric species, and our cluster analysis (Figure 1) and discriminant analysis (Table S1) supported the higher similarity of this group with the atmospherics. Low tank capacity was not, however, the only defining factor for this group, as some species listed with zero tank capacity (mostly derived from our predictive model) were grouped as shallow tanks. It is worth noting that the tank capacity formula we used was not precise enough to define shallow tank species, e.g., we obtained zero water holding capacity for *Wallisia anceps*, though the species’ trait values located it within the shallow tank group and a small tank capacity was confirmed by photographic evidence. The formula was, however, a useful approximation of the tank capacity for larger tanks and had a high predictive value (R^2^ = 0.81, df = 62, *p* <0.001; Figure 4).

The small tank capacity of the shallow tank group was accompanied by a decrease in stomatal density and A_max_, compared to the deep tank species (Figure 2). These are traits shared with the other atmospheric groups, which contribute to reduced transpiration water loss. Nevertheless, the group exhibited higher SLA compared to the pseudobulbs, potentially increasing leaf area for dew condensation. The range of tank volumes for shallow tanks of the species included in the cluster analysis was 2–60 mL, encompassing small-sized species such as *Tillandsia brachycaulos* with 5 mL tank capacity and larger-sized species such as *T. fasciculata* with 60 mL tank capacity. The dependance of these shallow tanks on dew was documented for *Tillandsia elongata* and *T. brachycaulos* in a dry forest in southern Mexico, particularly during the dry season [27,31]. *Tillandsia elongata*, with thinner leaves and lower trichome cover, was more efficient in dew condensation than *T. brachycaulos*, but both relied on this water source during the dry period. In contrast, the pseudobulbous species *Tillandsia yucatana* showed similar water loss in the presence or absence of dew. To date, information from other ecosystems as well as the level of dependency on dew vs. rain is lacking for other bromeliad species. High tank capacities are not needed for the small water volumes collected from dew. Low tank capacity may also be driven by reduced leaf area as a water saving strategy and a temperature regulation mechanism. Environmental requirements for the shallow tanks were intermediate between the pseudobulbs and the nebulophytes, being found at sites with lower precipitation and higher AI than the pseudobulbs, and thus supporting the importance of occult precipitation (as opposed to rain) for this group.

Finally, C_3_ tanks had lower adult plant size, and lower tank capacity, leaf thickness, water content per area, and trichome density, compared to CAM tanks. The combination of these traits conferred the C_3_ tanks less drought tolerance, compared to the CAM tanks, and, accordingly, C_3_ tanks were associated with wetter environments (higher precipitation, lower temperatures, and VPD) than the rest of the functional groups. Their distribution was similar to that of the CAM tanks, being mostly predominant in different tropical forests, but their distribution was associated with higher elevations than the CAM tanks. This climatic segregation (CAM tanks in lowlands and C_3_ tanks in highlands) has been observed along several forest altitudinal gradients [28,29,61].

Tropical rainforests and moist deciduous forests are high in epiphyte diversity and abundance [62,63], and in these forests all functional groups of epiphytic bromeliads converged (Figure 6). Despite being found in the same ecosystems, functional groups may segregate along microenvironmental gradients within each forest. Canopy vertical strata show large differences in light conditions and in temperature and air humidity, and epiphytes are unevenly distributed across these canopy gradients [17,24,25,31,64,65,66]. Shallow tanks and nebulophytes are found higher in the canopy, where they can cool faster and are more exposed to wind (carrying humidity). Their smaller size may also contribute to their ability to survive on small branches and even twigs in the outer forest canopy. In contrast, tree trunks and large branches are needed to support larger C_3_ or CAM tanks, which may in turn be more shade tolerant. Epiphyte functional traits exhibited little variation across broad-scale environmental gradients such as with altitude [44,47], instead displaying larger differences along local tree canopy gradients [47], and with host identity which also influenced epiphytic trait values [48]. These studies highlight the large microenvironmental ranges encountered within a single ecosystem, and that a shared habitat does not infer that functional groups thrive under the same climatic conditions.

The correlations between traits provided interesting insights into trait coordination and trade-offs, with leaf area and tank capacity being two important variables that modulate the interaction of epiphytic Bromeliaceae with their environment. Leaf area was calculated from maximum leaf width and length, with a very high predictive power based on the consistent leaf shape found in the family (intermediate between a triangular and rectangular shape). We used leaf area to estimate tank capacities within a reasonable margin of error and with a higher predictive power than previously published estimates using leaf width only [67]. Leaf length and width can be easily measured in herbarium specimens from public, digital images, which are available for many species. These formulae can better characterize species strategies for resource acquisition even though there is some error inherent in herbarium specimen measurements from shrinkage during the drying process.

Physiological traits are more difficult to obtain compared to anatomical traits, especially under field conditions. Thus, there is an underrepresentation of physiological traits in our dataset. A better coverage of physiological (“hard”) traits (*sensu* [68]) may have contributed to a better characterization of the functional groups. The few physiological proxies viz. traits, which included leaf δ^13^C and δ^15^N, and A_max_, provided significant insights, even when A_max_ was underrepresented among the species. Besides the difficulty of accessing the canopy with climbing equipment to measure photosynthesis, nocturnal gas exchange measurements in CAM species are often substituted by nocturnal acidity measures, complicating the comparison with C_3_ species. Differences in the units used for nocturnal acidity (fresh or dry weight or area based) further complicated our efforts to systematize these data in the current study. However, leaf δ^13^C provides a robust proxy of photosynthetic type (C_3_ versus CAM), of nocturnal CO_2_ uptake in CAM plants, and of the water-use efficiency in C_3_ plants [61].

Another relevant trait that was not included here was trichome type and size. Trichome density differences were observed among tank and atmospheric species but did not differentiate among the five groups (Figure 2). While similar in number, they may not be similar in trichome morphology or type, such as being hydrophilic or hydrophobic, and may result in different trichome covers deriving from differences in trichome size [41,42,43]. Efforts should be made to include more physiological variables and trichome traits in future studies, and to perform a better systematic evaluation of published data. 

We conclude that the five functional groups formed by unsupervised hierarchical cluster analysis, i.e., C_3_ tanks, CAM tanks, shallow tanks, pseudobulbs, and nebulophytes, provide a relevant overview of an array of strategies for water use/storage within the epiphytic Bromeliaceae. The cluster analysis provides quantifiable relationships among the previously described tank and atmospheric groups, and redefines the relationship of the species previously classified as tank-atmospheric intermediates (shallow tanks), classifying them closer to the atmospheric species and defining tank capacity for this group to range between 2 and 60 mL. The cluster analysis also provided support to separate nebulophytic species from pseudobulbs, based on the narrow leaf syndrome of the first group, which has been related to fog interception, and the high succulence degree of the latter group. These three atmospheric subgroups were also related to climatic variables, with decreasing dependance on rain and sensitivity to high temperatures and evapotranspiration in the nebulophytes, shallow tanks and pseudobulbs. C_3_ and CAM (deep) tanks were larger, had higher photosynthetic rates, and were more dependent on higher precipitation, with C_3_ species the most sensitive to drought and associated with higher elevation forests.

## 4. Materials and Methods

### 4.1. Trait Data

Fourteen traits were selected from the open access database compiled in Hietz et al. [6]. These data include records from adult individuals of epiphytic bromeliads from the whole American continent. We discarded all records that were not identified to the species level (we considered those with species aff. as valid records). These 14 selected traits had the highest record numbers for Bromeliaceae species. We added ten new traits that we considered important regarding the Bromeliaceae functional groups, which were tank water holding capacity (TC), leaf length (LL), maximal leaf width (LW), leaf index (LI=LL/LW), force to punch (FP), trichome density (TD), leaf water content per area (LWA), presence of pseudobulbs (PB), photosynthetic pathway (PP), and stomatal width (SW, Table 1). To compile these new variables, we took fresh measurements from available species, compiled published and unpublished data, or obtained values from digitized herbarium specimens and species descriptions (see references below). 

Trait measurements generally followed standardized methodologies [69] but may present variations of those. Among these are differences in the quantification of leaf dry matter content (LD; dry mass/water saturated fresh mass), which may differ in the method of leaf water saturation [6]. For leaf chlorophyll content per dry mass, we discarded data obtained through SPAD measurements, as the relationship between SPAD readings and leaf chlorophyll content may be highly variable among species with different leaf traits [70], which may be further complicated by the high differences in reflectance among epiphytic bromeliad leaves. Plant height, leaf width, and leaf length, when not available, were obtained from digital herbaria from the World Flora Online [71], from The Bromeliad Society of Australia image repository [72] and from the Chilean flora project plant database [73], or were measured from digitized herbarium images using ImageJ [74]. Herbarium images were downloaded from Tropicos [75], the Kew Royal Botanical Garden “Plants of the world online” collection [76], The Biodiversity Knowledge Integration Center from the University of Arizona [77], the Northeast Mexico Herbarium Network [78], and The National Herbarium of México open access collection [79]. Anatomic measurements such as leaf thickness, trichome density, and stomata size were obtained from published microscopic images that included a scale using ImageJ and from published and unpublished data (see Supplementary Materials, for a list of published studies used). Once the data were compiled, we deleted species that had only data for less than five traits. This left us with 16574 observations belonging to 204 species. Most of the data represent a single trait measurement of a species, though data from digital herbarium descriptions and from publications are averages of several measurements. In the case of species herbarium descriptions, we used maximum reported values of adult plant height, leaf width and leaf length. 

C_3_ and CAM photosynthetic pathways were assigned depending on leaf δ^13^C values, where those specimens with values –20‰ or higher were considered to belong to the CAM photosynthetic pathway [61]. When unavailable, leaf area (LA) was calculated from maximum leaf width and length. Considering that the shape of the bromeliad leaf is similar to a rectangle or a triangle, we tried both formulae, but a combination of these yielded estimates most similar to measured LA; therefore, the formula applied here was *LA* (cm^2^) = (LW*LL) / 1.5. Observed vs. calculated leaf areas were plotted for species in which LL, LW, and LA were measured in the same population or from the same herbarium image (n = 41 species), with the regression line having an R^2^ = 0.91 and a slope very close to one (m = 0.92), indicating a good predictive capacity (Figure 4a). Leaf area was calculated in 76% of the 204 species in the database.

Tank capacity was obtained from the literature (115 species; [80,81,82,83,84]) and measured in 36 further species available to the authors. Species that do not form tanks were assigned a tank capacity (TC) of 0 mL; for species with an unknown TC, we used LA to estimate TC, using the formula: *TC* (ml) = 0.0041 *LA*^2^ + 1.929 *LA −* 22.285, setting the origin to zero, providing an R^2^ = 0.81 for n = 63 species (Figure 4b). Tank capacity was calculated for 20% of the species, and only 4% for all TC values were calculated from estimated LA values. 

### 4.2. Gapfilling of Trait Data

In order to perform a hierarchical cluster analysis, we selected 76 species and 16 traits, for which the available data covered at least 70% of the species per trait and vice versa. Missing trait data represented 7.2% of the database for the 76 species. The R package Rphylopars [6,85] was used to impute missing data; this method assumes that the traits are correlated and phylogenetically inherited. A phylogenetic tree was constructed for the 50 species that were available in the R library V.PhyloMaker [86], providing a frame to impute the 47 missing data points. Three imputed values were deleted as they were outside the expected data range, and average values per genus were used to impute the 3% of the remaining missing data (in three cases trait values were left empty when no other species in the genus reference base was available for the imputation). 

### 4.3. Environmental Data

In order to relate functional groups to environmental variables of the species’ habitat, we downloaded environmental data from the open access database Global Biodiversity Information Facility [87] for all georeferenced records of the 76 species used in the cluster analysis. We complemented the database with georeferenced specimens from CICY and MEXU herbarium [79] and from Tropicos [75]. Data were collated to eliminate coordinates of species outside their natural habitat (e.g., botanical gardens or private collections). In the case of species with low record numbers, coordinates were approximated using collection site descriptions in the respective publications (references in Supplementary Materials).

Climatic environmental data from the years 1981 to 2010 were extracted from TerraClimate datasets [88]. Variables included vapor pressure deficit (VPD), precipitation (PPT), minimum temperature (T_min_), and maximum temperature (T_max_). Values of the global aridity index (AI) and of evapotranspiration (ET0) for the time period 1970 to 2000 were obtained from Zomer et al. [89]. We extracted additional environmental data, which included elevation (Elev), downloaded from WorldClim 2.1. [90], and 20 ecological zones, obtained from FAO [50]. Ecological zones (referred to from now on as ecosystems) are an approximate equivalent of the Köppen–Trewartha climatic types in combination with vegetation physiognomy and orography [50].

Distribution maps for each functional group were constructed by cartographically plotting the species’ coordinates on a base map of the American continent. Species coordinates were also cartographically overlaid on the environmental variables, for the estimation of the mean, minimum, and maximum values for each species record. In the case of the FAO [50] ecosystems, the respective categorical data were extracted for further analysis. All the above mapping approaches were carried out with QGis software version 191 3.6.3-Noosa [91].

### 4.4. Statistical Analyses

A two-way Ward’s hierarchical clustering method was performed using 16 traits (Table 1) and 76 species. The analysis provides both the multidimensional trait relationship between the species and the species variance within the trait space. Prior to the test, non-normal variables were log10 transformed to improve normality. Only nitrogen content and δ^15^N were not converted. Tank capacity data were converted by adding 0.5 (ml) to all the data in order to eliminate zero values. Absolute values of δ^13^C were used, inverting the most negative to the most positive δ^13^C values. Variables were also rescaled to obtain values between 0-1. A discriminant analysis (DA) was run to validate the significance of the formed functional groups using Squared Mahalanobis Distances between the groups. These analyses were performed in PAST 4.11 (cluster, [92]) and Statistica 13.5.0.17 (DA, Tibco Software Inc.).

Spearman Rank Order Correlations were calculated to evaluate the monotonic relationships between pairs of traits. Comparisons using Kruskal–Wallis tests and Wilcoxon rank sum post-hoc tests were used to evaluate significant differences in the traits (using raw values) between functional groups, and ANOVA and Tukey’s HSD post-hoc tests were used for normal distributed variables (leaf water content on area basis). These calculations were performed using the STAT 0.1.0 package [93] for R 4.0.2. [94]. We conducted Kruskal–Wallis tests and Wilcoxon rank sum post-hoc tests for each environmental variable, to determine differences in the environmental space inhabited by the functional groups.

For the ecological distribution of each functional group, we represented each species’ presence (not their abundance) per ecological zone, as abundance data may overrepresent the most widespread species and/or species inhabiting sites with higher sampling efforts. 

## Figures and Tables

**Table 1:** Functional trait units, abbreviations, medians, ranges, total number of records, and represented species and genera. Photosynthetic pathways = CAM, C_3_, CAM/C_3_. * Denotes variables used in hierarchical clustering.

Trait	Unit	Abbreviation	Median	Range	Records	Species	Genera
Adult plant height *	m	Height	0.4	(0.03–2.7)	507	126	16
Force to punch	N mm^−1^	FP	1.4	(0.17–6.2)	706	36	7
Leaf area *	cm^2^	LA	8678	(29–1033)	1702	201	23
Leaf carbon content per dry mass	%	C	45.2	(27.9–69.3)	502	59	11
Leaf carbon isotope signature *	‰	δ^13^C	−15.9	(−35.08–11)	903	197	23
Leaf chlorophyll content per dry mass	µg g^−1^	LCh	2800	(200–12200)	43	35	6
Leaf dry matter content	mg g	LD	161.2	(37.3–520)	1440	49	8
Leaf index *		LI	13.4	(2.2–250)	212	202	22
Leaf length *	cm	LL	38.7	(1.8–161)	539	204	23
Leaf nitrogen isotope signature *	‰	δ^15^N	−4.7	(−15.2–7.5)	643	69	12
Leaf nitrogen content per dry mass *	mg g	N	7.4	(1–25.5)	554	71	12
Leaf phosphorus content per dry mass	mg g	P	0.6	(0.02–5)	185	61	11
Leaf thickness *	mm	LT	0.6	(0.07–4.5)	1751	97	17
Leaf trichome density *	mm^−2^	TD	35.2	(2.8–120.9)	1104	74	11
Leaf water content on area basis *	g m^2^	LWA	540.8	(91.7–6017)	936	92	16
Leaf width *	cm	LW	3.0	(0.05–17.5)	449	202	22
Light saturated photosynthetic rate per leaf area	µmol m^2^ s	Amax	2.3	(0.7–4.7)	42	36	9
Specific leaf area *	mm^2^ mg^−1^	SLA	8.9	(0.01–68.5)	1709	109	16
Stomatal density *	mm^−2^	SD	21.0	(2.8–88.8)	975	102	14
Stomatal length	µm	SL	39.8	(14.4–284.3)	257	45	8
Stomatal width	µm	SW	35.0	(9.7–338.5)	56	32	7
Tank capacity *	ml	TC	85.7	(0–4924)	213	190	23
Total leaf water content *	g	LWC	3.1	(0.001–147)	738	92	16
Pseudobulb presence *		PB			204	204	23
Photosynthetic pathway					204	204	23
TOTAL					16,574	204	23

**Table 2:** Trait representation across genera of the Bromeliaceae family.

Genera	Records	Species	Traits
*Aechmea* Mez	1549	36	25
*Araeococcus* Brongn.	43	2	18
*Billbergia* Thunb.	280	8	22
*Canistropsis* (Mez) Leme	52	1	10
*Canistrum* E.Morren	18	2	8
*Catopsis* Griseb.	1138	7	25
*Fascicularia* Mez	78	1	11
*Goudaea* W.Till & Barfuss	15	1	10
*Guzmania* Ruiz & Pav.	2069	31	25
*Josemania* W.Till & Barfuss	13	1	12
*Lemeltonia* Barfuss & W.Till	206	1	21
*Lutheria* Barfuss & W.Till	21	1	18
*Lymania* Read	10	1	9
*Mezobromelia* L. B. Sm.	9	1	9
*Neoregelia* L. B. Sm.	63	5	14
*Nidularium* Lem.	92	8	19
*Quesnelia* Gaudich.	21	2	11
*Racinaea* M.A.Spencer & L.B.Sm.	878	5	22
*Ronnbergia* E.Morren & Andre	18	2	8
*Tillandsia* L.	9522	81	25
*Vriesea* Lindl.	93	5	14
*Wallisia* E.Morr.	411	1	24
*Werauhia* J.R.Grant.	75	6	19

**Table 3:** Functional groups, description of the main traits that define them, main water source, photosynthetic pathway, genera, and corresponding previous classifications. Water sources information was taken from previous studies; genera are listed from higher to lower abundance according to the species analyzed in this study. Previous classification refers to (P) Pittendrigh [17] and (B) Benzing [21].

Functional Group	Description	Main Water Source	C_3_ or CAM	Genera	Previous Classification
Nebulophytes	Acicular leaves, usually with high leaf index, no tank capacity	Fog	Mostly CAM	*Tillandsia*, *Araeococcus*	(P) Type IV Atmosphere-Absorbing trichome (B) Type V
Pseudobulbs	Neotenic, forming pseudobulbs, highly succulent, thick leaves, no tank capacity	Rain/ internal reserves	CAM	*Tillandsia*	(P) Type IV Atmosphere-Absorbing trichome (B) Type V
Shallow tanks	2–60 mL tank capacity, small sized, thin leaves	Dew/rain	Mostly CAM	*Tillandsia*, *Araeococcus*, *Aechmea*, *Wallisia*, *Lemeltonia*, *Canistrum*, *Neoregelia*, *Nidularium*, *Billbergia*, *Quesnelia*, *Ronnbergia*	(P) Type IV Atmosphere-Absorbing trichome (B) Type V
CAM tank	>61 mL tank capacity, large size	Rain	CAM	*Aechmea*, *Tillandsia*, *Billbergia*, *Neoregelia*, *Nidularium*, *Canistropsis*, *Canistrum*, *Lymania*, *Quesnelia*	(P) Type III Tank-Absorbing trichome (B)Type III
C_3_ tank	>5 mL tank capacity, low specific leaf area, medium to large size	Rain	C_3_	*Tillandsia*, *Catopsis*, *Guzmania*, *Billbergia*, *Lutheria*, *Werauhia*, *Racinaeae*, *Fascicularia*, *Goudaea*, *Josemania*, *Mesobromelia*, *Vriesea*	(P) Type III Tank-Absorbing trichome (B)Type IV

## Data Availability

The datasets analyzed during the current study are available at: Functional traits previously published, https://doi.org/10.5061/dryad.7wm37pvtf; Functional and climatic traits generated, https://doi.org/10.5281/zenodo.7101921.

## Supplementary Materials

The following supporting information can be downloaded at: https://www.mdpi.com/article/10.3390/plants11223151/s1, Table S1: Tests of Significance of Squared Mahalanobis Distances for the Discriminant Analysis; Table S2: Species functional group affiliation, number of records and number of traits represented within the trait database; Table S3: Spearman rank order correlations among traits; Figure S1: Comparison of functional traits between five epiphytic Bromeliaceae functional groups; References [95,96,97,98,99,100,101,102,103,104,105,106,107,108,109,110,111,112,113,114,115,116,117,118,119,120,121,122,123,124,125,126,127,128,129,130,131,132,133,134,135,136,137,138,139,140,141,142,143,144,145,146,147,148,149,150,151,152,153,154,155,156,157,158,159,160,161] are cited in Supplementary Materials.
