# Supplementary material for: New Proposal of Epiphytic Bromeliaceae Functional Groups to Include Nebulophytes and Shallow Tanks
Source: Plants (Basel). 2022 Nov 17;11(22):3151. doi: 10.3390/plants11223151 (PMC9693514; doi:10.3390/plants11223151)
Supplement: Supplementary file 1 [file plants-11-03151-s001.zip › plants-1967419-supplementary.pdf]

**Supplementary Materials**

# New proposal of epiphytic Bromeliaceae functional groups to include nebulophytes and shallow tanks

# New Proposal of Epiphytic Bromeliaceae Functional Groups to Include Nebulophytes and Shallow Tanks

**Casandra Reyes-García [1,*], Narcy Anai Pereira-Zaldívar [1], Celene Espadas-Manrique [1], Manuela Tamayo-Chim [1], Nahlleli Chilpa-Galván [1], Manuel Jesús Cach-Pérez [2], Marypaz Ramírez-Medina [1], Ana Maria Benavides [3], Peter Hietz [4], Gerhard Zotz [5,6], José Luis Andrade [1], Catherine Cardelús [7], Rodolfo de Paula Oliveira [8], Helena J. R. Einzmann [5], Valeria Guzmán Jacob [9], Thorsten Krömer [10], Juan P. Pinzón [11], Juliano Sarmento Cabral [12], Wolfgang Wanek [13] and Carrie Woods [14]**

1 Unidad de Recursos Naturales, Centro de Investigación Científica de Yucatán, 43 St., Chuburna de Hidalgo, Mérida 97205, Mexico
2 El Colegio de la Frontera Sur, Guineo, Second Section, Villahermosa 86280, Mexico
3 Jardín Botánico de Medellín, 73 St., Medellín 50035, Colombia
4 Department of Integrative Biology and Biodiversity Research, University of Natural Resources and Life Sciences, Gregor-Mendel-Straße 33, 1180 Vienna, Austria
5 Functional Ecology Group, Institute of Biology and Environmental Sciences, University of Oldenburg, Box 2503, D-26111 Oldenburg, Germany
6 Smithsonian Tropical Research Institute, Panama 32402, Panama
7 Departments of Biology and Environmental Studies, Colgate University, 13 Oak Dr E Ext, Hamilton, NY 13346, USA
8 Departament of Botany, University of Brasilia, Brasilia 70910-900, Brazil
9 Biodiversity, Macroecology and Biogeography, University of Goettingen, 37073 Göttingen, Germany
10 Centro de Investigaciones Tropicales, Universidad Veracruzana, Veracruz 91090, Mexico
11 Departamento de Botánica, Campus de Ciencias Biológicas y Agropecuarias, Universidad Autónoma de Yucatán, Road Mérida-Xmatkuil km 15.5, Mérida 97315, Mexico
12 School of Biosciences, University of Birmingham, Edgbaston B15 2TT, Birmingham, UK
13 Center of Microbiology and Environmental Systems Science, University of Vienna, Djerassipl, 1030 Vienna, Austria
14 Department of Biology, University of Puget Sound. 1500 N Warner St, Tacoma, WA 98416, USA
* Correspondence: creyes@cicy.mx; Tel.: +(52)-999-942-83-30

**Table S1**. Tests of Significance of Squared Mahalanobis Distances for the Discriminant Analysis. F tests with 15 and 55 degrees of freedom. Dist. represents the Mahalanobis distance between the groups. **p<0.001.

| | CAM tank | | $C_3$ tank | | Shallow tank | | Pseudobulbs | | Nebulophyte | |
|---|---|---|---|---|---|---|---|---|---|---|
| Class | F | Dist. | F | Dist. | F | Dist. | F | Dist. | F | Dist. |
| CAM tank | | 0.0 | 10.7 | 20.8** | 13.2 | 31.5** | 20.6 | 64.6** | 24.5 | 64.0** |
| $C_3$ tank | 10.7 | 20.8** | | 0.0 | 13.4 | 30.1** | 22.7 | 67.8** | 26.5 | 65.3** |
| Shallow tank | 13.2 | 31.5** | 13.4 | 30.1** | | 0.0 | 3.6 | 12.4** | 6.2 | 18.2** |
| Pseudobulbs | 20.6 | 64.6** | 22.7 | 67.8** | 3.6 | 12.3** | | 0.0 | 5.5 | 20.2** |
| Nebulophyte | 24.5 | 64.0** | 26.5 | 65.3** | 6.2 | 18.2** | 5.5 | 20.2** | | 0.0 |

**Table S2**. Species functional group affiliation, number of records and number of traits represented within the trait database.

| **Species** | **Records** | **Traits** | **Functional group** |
|---|---|---|---|
| *Aechmea abbreviata L.B. Sm.* | 14 | 9 | Shallow tank |
| *Aechmea angustifolia Poepp. & Endl.* | 14 | 12 | Shallow tank |
| *Aechmea aquilega (Salisb.) Griseb.* | 138 | 19 | CAM tank |
| *Aechmea bracteata (Sw.) Griseb.* | 263 | 21 | CAM tank |
| *Aechmea brevicollis L. B. Sm.* | 18 | 9 | Shallow tank |
| *Aechmea bromeliifolia (Rudge) Baker* | 191 | 22 | CAM tank |
| *Aechmea castelnavii Baker.* | 9 | 8 | CAM tank |
| *Aechmea chantinii (Carr.) Baker* | 12 | 12 | CAM tank |
| *Aechmea cucullata H. Luther.* | 10 | 9 | CAM tank |
| *Aechmea dichlamydea Baker* | 33 | 15 | CAM tank |
| *Aechmea echinata (Leme) Leme.* | 9 | 8 | CAM tank |
| *Aechmea farinosa (Regel) L. B. Sm.* | 9 | 8 | Shallow tank |
| *Aechmea fasciata* (Lindl.) Baker | 13 | 13 | CAM tank |
| *Aechmea fendleri André ex Mez.* | 73 | 18 | CAM tank |
| *Aechmea filicaulis (Griseb.) Mez.* | 17 | 15 | CAM tank |
| *Aechmea gamosepala* Wittm. | 13 | 13 | CAM tank |
| *Aechmea gurkeniana E. Pereira & Moutinho.* | 9 | 8 | CAM tank |
| *Aechmea hoppii (Harms) L. B. Sm.* | 9 | 8 | CAM tank |
| *Aechmea lingulata (L.) Baker* | 33 | 15 | CAM tank |
| *Aechmea manzanaresiana H. Luther* | 9 | 8 | CAM tank |
| *Aechmea mariae-reginae H. Wendl.* | 9 | 8 | CAM tank |

| | | | |
|---|---|---|---|
| *Aechmea mertensii (G.Mey.) Schult. & Schult. f.* | 37 | 19 | CAM tank |
| *Aechmea milsteiniana L. B. Sm. & Read.* | 9 | 8 | CAM tank |
| *Aechmea nallyi L. B. Sm.* | 9 | 8 | CAM tank |
| *Aechmea nudicaulis Griseb.* | 229 | 22 | CAM tank |
| *Aechmea paniculigera (Sw.) Griseb.* | 12 | 12 | CAM tank |
| *Aechmea pubescens Baker.* | 14 | 12 | CAM tank |
| *Aechmea racinae L. B. Sm.* | 10 | 9 | Shallow tank |
| *Aechmea rodriguesiana (L. B. Sm.) L. B. Sm.* | 14 | 13 | CAM tank |
| *Aechmea servitensis André.* | 175 | 14 | Shallow tank |
| *Aechmea setigera Mart. ex Schult. & Schult. f.* | 23 | 19 | CAM tank |
| *Aechmea tillandsioides (Mart. ex Schult. & Schult. f.) Baker* | 77 | 21 | CAM tank |
| *Aechmea tomentosa Mez.* | 5 | 5 | CAM tank |
| *Aechmea weilbachii Didr.* | 20 | 10 | CAM tank |
| *Aechmea woronowii Harms* | 9 | 8 | CAM tank |
| *Araeococcus micranthus Brongn* | 32 | 15 | Shallow tank |
| *Araeococcus pectinatus L. B. Sm.* | 12 | 11 | Nebulophyte |
| *Billbergia amoena (Lodd.) Lindl.* | 21 | 17 | CAM tank |
| *Billbergia euphemiae E. Morren.* | 16 | 15 | $C_3$ tank |
| *Billbergia manarae Steyerm.* | 12 | 11 | CAM tank |
| *Billbergia meyeri Mez.* | 9 | 8 | Shallow tank |
| *Billbergia porteana Brongniart ex Beer* | 185 | 20 | CAM tank |
| *Billbergia sanderiana E. Morren* | 12 | 11 | CAM tank |
| *Billbergia stenopetala Harms* | 12 | 11 | CAM tank |
| *Billbergia zebrina (Herbert) Lindl.* | 13 | 12 | CAM tank |
| *Canistropsis billbergioides (Schult. & Schult. f.) Leme.* | 52 | 10 | CAM tank |
| *Canistrum lanigerum H. Luther & Leme.* | 9 | 8 | CAM tank |
| *Canistrum seidelianum W. Weber* | 9 | 8 | Shallow tank |
| *Catopsis berteroniana (Schult. & Schult. f.) Mez* | 175 | 23 | $C_3$ tank |
| *Catopsis floribunda (Brongn.) L. B. Smith.* | 21 | 19 | $C_3$ tank |
| *Catopsis juncifolia Mez & Wercklé.* | 16 | 15 | $C_3$ tank |
| *Catopsis morreniana Mez* | 140 | 21 | $C_3$ tank |
| *Catopsis nitida (Hook.) Griseb.* | 177 | 20 | $C_3$ tank |
| *Catopsis nutans (Sw.) Griseb.* | 34 | 16 | $C_3$ tank |

| | | | |
|---|---|---|---|
| *Catopsis sessiliflora (Ruiz & Pav.) Mez.* | 576 | 22 | $C_3$ tank |
| *Fascicularia bicolor (Ruiz & Pav.) Mez.* | 78 | 11 | $C_3$ tank |
| *Goudea ospinae (H.Luther) W.Till & Barfuss* | 15 | 10 | $C_3$ tank |
| *Guzmania altsonii L. B. Sm.* | 9 | 8 | $C_3$ tank |
| *Guzmania angustifolia (Baker) Wittm.* | 24 | 14 | $C_3$ tank |
| *Guzmania blassii Rauh* | 9 | 8 | $C_3$ tank |
| *Guzmania calothyrsus Mez* | 9 | 8 | $C_3$ tank |
| *Guzmania desautelsii Read & L. B. Sm.* | 17 | 11 | $C_3$ tank |
| *Guzmania dissitiflora (André) L. B. Sm.* | 9 | 8 | $C_3$ tank |
| *Guzmania donnellsmithii Mez ex Donn. Sm.* | 26 | 13 | $C_3$ tank |
| *Guzmania eduardii André ex Mez* | 9 | 8 | $C_3$ tank |
| *Guzmania fosteriana L. B. Sm.* | 144 | 15 | $C_3$ tank |
| *Guzmania glomerata Mez & Wercklé.* | 13 | 10 | $C_3$ tank |
| *Guzmania laeta H. E. Luther* | 161 | 14 | $C_3$ tank |
| *Guzmania lingulata (L. ) Mez* | 409 | 25 | $C_3$ tank |
| *Guzmania megastachya (Baker) Mez* | 33 | 15 | $C_3$ tank |
| *Guzmania melinonis Regel* | 9 | 8 | $C_3$ tank |
| *Guzmania mitis L. B. Sm.* | 155 | 16 | $C_3$ tank |
| *Guzmania monostachia (L.) Rusby ex Mez* | 223 | 23 | $C_3$ tank |
| *Guzmania musaica (Linden & André) Mez* | 16 | 14 | $C_3$ tank |
| *Guzmania nicaraguensis Mez & C. F. Baker* | 9 | 8 | $C_3$ tank |
| *Guzmania osyana (E. Morren) Mez* | 9 | 8 | $C_3$ tank |
| *Guzmania patula Mez & Wercklé* | 140 | 16 | $C_3$ tank |
| *Guzmania plicatifolia L. B. Sm.* | 9 | 8 | $C_3$ tank |
| *Guzmania rauhiana H. E. Luther* | 9 | 8 | $C_3$ tank |
| *Guzmania remyi L. B. Sm.* | 10 | 9 | $C_3$ tank |
| *Guzmania retusa L. B. Sm.* | 16 | 13 | $C_3$ tank |
| *Guzmania sanguinea (André) André ex Mez* | 14 | 13 | $C_3$ tank |
| *Guzmania scherzeriana Mez* | 16 | 11 | $C_3$ tank |
| *Guzmania subcorymbosa L. B. Sm.* | 125 | 19 | $C_3$ tank |
| *Guzmania tarapotina Ule* | 9 | 8 | $C_3$ tank |
| *Guzmania triangularis L. B. Sm.* | 296 | 14 | $C_3$ tank |
| *Guzmania wittmackii (André) André ex Mez* | 13 | 12 | $C_3$ tank |
| *Guzmania zahnii (Hook. f.) Mez* | 19 | 10 | $C_3$ tank |

| | | | |
|---|---|---|---|
| *Josemania singularis (Mez & Wercklé) W.Till & Barfuss* | 13 | 12 | $C_3$ tank |
| *Lemeltonia monadelpha (E.Morren) Barfuss W. & Till* | 206 | 21 | Shallow tank |
| *Lutheria splendens (Brongn.) Barfuss & W.Till* | 21 | 18 | $C_3$ tank |
| *Lymania globosa Leme* | 10 | 9 | CAM tank |
| *Mezobromelia pleiosticha (Griseb.) Utley & H. Luther* | 12 | 11 | $C_3$ tank |
| *Neoregelia carolinae (Beer) L. B. Sm.* | 19 | 15 | CAM tank |
| *Neoregelia chlorosticta (É. Morren) L. B. Sm.* | 12 | 11 | CAM tank |
| *Neoregelia eltoniana W. Weber* | 9 | 8 | CAM tank |
| *Neoregelia gavionensis* Martinelli & Leme | 12 | 11 | CAM tank |
| *Neoregelia nivea Leme* | 12 | 11 | Shallow tank |
| *Nidularium bicolor (E. Pereira) Leme* | 9 | 8 | CAM tank |
| *Nidularium billbergioides (Schult. & Schult. f.) L. B. Sm.* | 12 | 12 | Shallow tank |
| *Nidularium fulgens Lemaire* | 14 | 13 | CAM tank |
| *Nidularium innocentii Lemaire* | 16 | 14 | $C_3$ tank |
| *Nidularium procerum Lindman* | 19 | 17 | CAM tank |
| *Nidularium purpureum Beer* | 10 | 9 | CAM tank |
| *Nidularium rutilans E. Morren* | 11 | 9 | CAM tank |
| *Quesnelia imbricata L. B. Sm.* | 9 | 8 | Shallow tank |
| *Quesnelia marmorata (Lemaire) R. W. Read* | 12 | 11 | CAM tank |
| *Racinaea adpressa (André) J. R. Grant* | 298 | 14 | $C_3$ tank |
| *Racinaea contorta (Mez & Pittier ex Mez) M. A. Spencer & L. B. Sm.* | 162 | 18 | $C_3$ tank |
| *Racinaea dyeriana (André) Barfuss & W.Till* | 8 | 8 | $C_3$ tank |
| *Racinaea ghiesbreghtii (Baker) M.A.Spencer & L.B.Sm.* | 96 | 18 | $C_3$ tank |
| *Racinaea tetrantha (Ruiz & Pav.) M. A. Spencer & L. B. Sm.* | 315 | 14 | $C_3$ tank |
| *Ronnbergia columbiana E. Morren* | 9 | 8 | Shallow tank |
| *Ronnbergia tonduzii (Mez & Pittier) Aguirre-Santoro* | 9 | 8 | Shallow tank |
| *Tillandsia albida* Mez & Purpus | 24 | 15 | Shallow tank |
| *Tillandsia australis Mez* | 12 | 11 | $C_3$ tank |
| *Tillandsia baileyi Rose ex Small* | 15 | 14 | Pseudobulbs |

| | | | |
|---|---|---|---|
| *Tillandsia balbisiana Schult. & Schult. f.* | 264 | 23 | Pseudobulbs |
| *Tillandsia bartramii Elliott* | 9 | 9 | Nebulophyte |
| *Tillandsia bergeri Mez* | 12 | 12 | Nebulophyte |
| *Tillandsia biflora Ruiz & Pav.* | 149 | 15 | $C_3$ tank |
| *Tillandsia brachycaulos Schltdl.* | 576 | 22 | Shallow tank |
| *Tillandsia bulbosa Hook* | 265 | 25 | Pseudobulbs |
| *Tillandsia butzii* Mez | 270 | 21 | Pseudobulbs |
| *Tillandsia capitata Griseb* | 9 | 9 | Shallow tank |
| *Tillandsia caput-medusae E. Morren* | 19 | 16 | Pseudobulbs |
| *Tillandsia carlsoniae L. B. Sm.* | 9 | 8 | CAM tank |
| *Tillandsia chaetophylla Mez* | 8 | 8 | Nebulophyte |
| *Tillandsia circinnata Schltdl.* | 17 | 9 | Pseudobulbs |
| *Tillandsia compacta Griseb* | 10 | 10 | $C_3$ tank |
| *Tillandsia complanata Benth* | 341 | 22 | $C_3$ tank |
| *Tillandsia concolor L. B. Sm.* | 300 | 20 | Shallow tank |
| *Tillandsia copanensis Rauh & Rutschm.* | 12 | 11 | CAM tank |
| *Tillandsia dasyliriifolia Baker* | 262 | 17 | CAM tank |
| *Tillandsia deppeana Steud* | 40 | 16 | $C_3$ tank |
| *Tillandsia eistetteri Ehlers* | 124 | 15 | Nebulophyte |
| *Tillandsia elongata Kunth* | 300 | 20 | Shallow tank |
| *Tillandsia excelsa Griseb* | 14 | 12 | $C_3$ tank |
| *Tillandsia exserta Mez* | 9 | 8 | Shallow tank |
| *Tillandsia fasciculata Sw.* | 531 | 25 | Shallow tank |
| *Tillandsia festucoides Brongn. ex Mez* | 93 | 19 | Nebulophyte |
| *Tillandsia filifolia Schltdl. & Cham.* | 142 | 18 | Nebulophyte |
| *Tillandsia flagellata L. B. Sm.* | 10 | 9 | Shallow tank |
| *Tillandsia flexuosa Sw.* | 376 | 20 | Shallow tank |
| *Tillandsia funckiana Baker* | 24 | 15 | Nebulophyte |
| *Tillandsia gardneri* Lindl. | 14 | 12 | CAM tank |
| *Tillandsia gymnobotrya Baker* | 12 | 10 | $C_3$ tank |
| *Tillandsia hammeri Rauh & Ehlers* | 8 | 8 | Nebulophyte |
| *Tillandsia heterophylla E. Morren* | 16 | 12 | $C_3$ tank |
| *Tillandsia imperialis E. Morren ex Roezl* | 17 | 15 | $C_3$ tank |
| *Tillandsia intermedia Mez* | 149 | 16 | Pseudobulbs |
| *Tillandsia ionantha Planch.* | 379 | 24 | Nebulophyte |
| *Tillandsia juncea (Ruiz & Pav.) Poir.* | 511 | 23 | Nebulophyte |
| *Tillandsia kirchhoffiana Wittm.* | 197 | 18 | $C_3$ tank |
| *Tillandsia krukoffiana L. B. Sm.* | 12 | 11 | CAM tank |
| *Tillandsia landbeckii Phil.* | 36 | 15 | Nebulophyte |
| *Tillandsia latifolia Meyen* | 17 | 13 | Shallow tank |

| | | | |
|---|---|---|---|
| *Tillandsia leiboldiana Schltdl.* | 20 | 18 | $C_3$ tank |
| *Tillandsia limbata Schltdl.* | 14 | 12 | CAM tank |
| *Tillandsia lucida E.Morren ex Baker* | 12 | 11 | $C_3$ tank |
| *Tillandsia lymanii Rauh* | 12 | 11 | CAM tank |
| *Tillandsia makoyana Baker* | 126 | 16 | CAM tank |
| *Tillandsia multicaulis Steud.* | 334 | 20 | $C_3$ tank |
| *Tillandsia paleacea C. Presl* | 20 | 17 | Nebulophyte |
| *Tillandsia paucifolia* Baker | 21 | 19 | Pseudobulbs |
| *Tillandsia pentasticha Rauh & Wülfingh.* | 8 | 8 | Nebulophyte |
| *Tillandsia polystachia (L.) L.* | 557 | 24 | Shallow tank |
| *Tillandsia pruinosa* Sw. | 21 | 18 | Pseudobulbs |
| *Tillandsia pseudosetacea Ehlers & Rauh* | 9 | 9 | Nebulophyte |
| *Tillandsia punctulata Schltdl. & Cham.* | 361 | 20 | $C_3$ tank |
| *Tillandsia recurvata (L.) L.* | 196 | 22 | Nebulophyte |
| *Tillandsia rhomboidea André* | 9 | 8 | $C_3$ tank |
| *Tillandsia rodrigueziana Mez* | 9 | 8 | Shallow tank |
| *Tillandsia roland-gosselinii Mez* | 9 | 8 | CAM tank |
| *Tillandsia rothii Rauh* | 133 | 15 | Shallow tank |
| *Tillandsia rotundata (L. B. Sm.) C. S. Gardner* | 9 | 8 | CAM tank |
| *Tillandsia schiedeana* Steud. | 326 | 22 | Nebulophyte |
| *Tillandsia sessemocinoi López-Ferr., Espejo & P.Blanco* | 7 | 7 | Nebulophyte |
| *Tillandsia setacea* Sw. | 11 | 11 | Nebulophyte |
| *Tillandsia setiformis Ehlers* | 8 | 8 | Nebulophyte |
| *Tillandsia streptophylla Scheidw. ex E. Morren* | 446 | 21 | Shallow tank |
| *Tillandsia stricta Sol. ex Ker Gawl.* | 26 | 19 | Nebulophyte |
| *Tillandsia subulifera Mez* | 35 | 15 | Pseudobulbs |
| *Tillandsia suescana L. B. Sm.* | 11 | 10 | $C_3$ tank |
| *Tillandsia tenuifolia L.* | 195 | 20 | Shallow tank |
| *Tillandsia tovarensis Mez* | 170 | 19 | $C_3$ tank |
| *Tillandsia tricolor Schltdl. & Cham.* | 69 | 20 | Shallow tank |
| *Tillandsia usneoides (L.) L.* | 97 | 23 | Nebulophyte |
| *Tillandsia utriculata L.* | 506 | 24 | CAM tank |
| *Tillandsia variabilis Schltdl.* | 11 | 11 | Shallow tank |
| *Tillandsia venusta Mez & Wercklé* | 43 | 19 | $C_3$ tank |
| *Tillandsia xerographica Rohweder* | 15 | 13 | CAM tank |
| *Tillandsia yucatana Baker* | 50 | 16 | Pseudobulbs |
| *Vriesea fenestralis* Linden & André | 12 | 12 | $C_3$ tank |
| *Vriesea fosteriana* L. B. Sm. | 12 | 12 | $C_3$ tank |

| | | | |
|---|---|---|---|
| *Vriesea guttata* Linden & André | 11 | 11 | $C_3$ tank |
| *Vriesea neoglutinosa* Mez | 31 | 14 | $C_3$ tank |
| *Vriesea zamorensis (L. B. Sm.) L. B. Sm.* | 27 | 10 | $C_3$ tank |
| *Wallisia anceps (G.Lodd.) Barfuss & W.Till* | 411 | 24 | Shallow tank |
| *Werauhia gigantea* (Mart. ex Schult. f.) J. R. Grant | 11 | 11 | $C_3$ tank |
| *Werauhia lutheri S. Pierce & Aranda.* | 14 | 9 | $C_3$ tank |
| *Werauhia marnier-lapostollei (L. B. Sm.) J. R. Grant* | 13 | 13 | $C_3$ tank |
| *Werauhia sanguinolenta (Cogn. & Marchal) J. R. Grant* | 22 | 18 | $C_3$ tank |
| *Werauhia viridiflora (Regel) J. R. Grant* | 12 | 11 | $C_3$ tank |

**Table S3.** Spearman rank order correlations. Height=adult plant height, FP=force to punch, LA=leaf area, LD=leaf dry matter content, SLA=specific leaf area, $\delta^{13}C$= leaf carbon isotope signature, C=leaf carbon content per leaf dry mass, LCh=leaf chlorophyll content per leaf dry mass, LI=leaf index (leaf length/leaf width), LL=leaf length, $\delta^{15}N$= leaf nitrogen isotope signature, N=leaf nitrogen content per leaf dry mass, P=leaf phosphorus content per leaf dry mass, LT=leaf thickness, TD=leaf trichomes density, LWC=total leaf water content, LW=leaf width, $A_{max}$=light saturated photosynthetic rate per leaf area, SD=abaxial stomatal density, SL=stomatal length, SW=stomatal width, LWA=leaf water content on area basis, TC=tank capacity. Upper diagonal show Spearman correlations, lower diagonal shows p value, p >0.05.

| | TC | LWA | SW | SL | SD | $A_{max}$ | LW | LWC | TD | LT | P | N | $\delta^{15}N$ | LL | LI | LD | LCh | C | $\delta^{13}C$ | SLA | LA | FP | Height |
|---|---|---|---|---|---|---|---|---|---|---|---|---|---|---|---|---|---|---|---|---|---|---|---|
| **Height** | 5.30E-01 | -1.20E-01 | -2 E-01 | 4.20E-04 | 5.40E-01 | 5.58E-01 | 5.71E-01 | 5.70E-01 | -2.52E-01 | -7.10E-02 | -1.50E-01 | -7.11E-02 | 3.38E-01 | 6.25E-01 | -1.93E-01 | 3.47E-01 | -6.09E-02 | 1.11E-01 | -9.20E-02 | 7.50E-02 | 6.20E-01 | 3.30E-01 | |
| **FP** | 1.50E-01 | -1.70E-02 | -2.8E-01 | -8.60E-02 | 3.90E-01 | 4.42E-01 | 2.59E-01 | 2.50E-01 | 2.43E-01 | -2 E-01 | 4.90E-02 | -3.36E-02 | 3.58E-01 | 4.72E-01 | 1.03E-01 | -5.20E-02 | 3.08E-01 | 5.61E-01 | -2.60E-01 | -1.70E-01 | 2.20E-01 | | 7 E-02 |
| **LA** | 8.40E-01 | -1.50E-01 | -8.40E-02 | 1.40E-01 | 5.60E-01 | 6.63E-01 | 8.50E-01 | 9.24E-01 | -4.15E-01 | -1.3E-01 | -5.70E-02 | 2.57E-02 | 4.30E-01 | 6.69E-01 | -4.47E-01 | 3.42E-01 | -2.80E-01 | 4.09E-02 | -5.40E-02 | 5.40E-02 | | 2.00E-01 | 7.40E-15 |
| **SLA** | 1.70E-01 | -6.30E-01 | -1.10E-01 | 1.30E-02 | 2.40E-01 | 6.25E-01 | 6.87E-02 | -1.65E-01 | -3.15E-01 | -5.70E-01 | 1.00E-01 | 1.47E-01 | 2.93E-01 | 1.25E-02 | -1.00E-01 | -2.91E-02 | -1.75E-02 | 2.50E-01 | -4.60E-01 | | 5.70E-01 | 3.30E-01 | 5.00E-01 |
| **$\delta^{13}C$** | -7.90E-02 | 3.10E-01 | -4.50E-02 | -3.60E-01 | -2 E-01 | -5.94E-01 | -8.34E-02 | 1.04E-01 | 2.35E-01 | 5.30E-01 | -3.20E-02 | -2.01E-01 | -3.60E-01 | 2.02E-02 | 7.96E-02 | -1.25E-01 | -4.75E-01 | -4.06E-01 | | 5.80E-07 | 4.50E-01 | 1.23E-01 | 3.20E-01 |
| **C** | 9.70E-02 | -2.20E-01 | -5.90E-01 | 9.70E-02 | 3.9E-01 | 2.88E-01 | 7.91E-02 | 1.52E-02 | -2.67E-02 | -3.40E-01 | -1.60E-02 | 1.43E-01 | 2.56E-01 | 1.20E-01 | -3.03E-02 | 3.87E-01 | 4.36E-01 | | 1.42E-03 | 9.80E-02 | 7.60E-01 | 6.64E-03 | 4.20E-01 |
| **LCh** | -2.30E-01 | -2.80E-01 | 5.70E-02 | 2.40E-01 | -8.30E-02 | 5.38E-01 | -1.62E-01 | -3.50E-01 | 4.63E-02 | -2.60E-01 | -6.80E-02 | 4.05E-01 | 1.34E-01 | -1.70E-01 | 1.70E-01 | -3.04E-01 | | 3.76E-02 | 3.93E-03 | 9.30E-01 | 1.00E-01 | 2.14E-01 | 7.40E-01 |
| **LD** | 3.60E-01 | -1.80E-01 | -1.80E-01 | 1.10E-01 | 3.50E-01 | -4.40E-02 | 1.09E-01 | 4.20E-02 | 1.26E-01 | -3.40E-01 | -3.90E-01 | -1.10E-01 | 1.30E-01 | 2.29E-01 | -2.24E-02 | | 2.19E-01 | 2.37E-02 | 3.92E-01 | 8.40E-01 | 1.60E-02 | 7.93E-01 | 2.10E-02 |
| **LI** | -4.40E-01 | 9.80E-02 | -4.20E-03 | 1.40E-01 | -5.10E-02 | -4.88E-01 | -7.12E-01 | -5.12E-01 | 3.12E-01 | 1.20E-01 | 1.30E-01 | 7.21E-02 | -3.30E-01 | 1.20E-01 | | 8.79E-01 | 3.28E-01 | 8.20E-01 | 2.68E-01 | 3 E-01 | 3.00E-11 | 5.51E-01 | 3 E-02 |
| **LL** | 5.20E-01 | -1.30E-01 | -1.80E-01 | 2.10E-01 | 4.60E-01 | 3.71E-01 | 5.01E-01 | 6.81E-01 | -1.61E-01 | -8.60E-02 | -2.10E-02 | -5.96E-02 | 3.09E-01 | | 8.97E-02 | 1.13E-01 | 3.29E-01 | 3.66E-01 | 7.79E-01 | 9 E-01 | 1.80E-27 | 3.67E-03 | 5.30E-15 |
| **$\delta^{15}N$** | 5.10E-01 | -2.30E-01 | -5.40E-01 | -2.00E-01 | 4.80E-01 | 7.07E-01 | 4.54E-01 | 4.00E-01 | -3.11E-01 | -2.10E-01 | 3.20E-02 | 2.23E-01 | | 9.80E-03 | 5.05E-03 | 4.49E-01 | 5.31E-01 | 4.99E-02 | 2.71E-03 | 3.20E-02 | 2.30E-04 | 8.56E-02 | 6 E-03 |
| **N** | 2.10E-03 | -4.40E-02 | -1.10E-01 | -2 E-01 | 1.80E-01 | 2.25E-01 | -5.37E-02 | 7.07E-02 | -1.99E-01 | -1.60E-01 | 1.30E-01 | | 7.39E-02 | 6.22E-01 | 5.50E-01 | 5.31E-01 | 4.00E-02 | 2.80E-01 | 9.60E-02 | 2.80E-01 | 8.30E-01 | 8.79E-01 | 5.70E-01 |
| **P** | -2.10E-03 | 2 E-01 | -2.30E-01 | -2.6E-01 | -1.10E-02 | -1.49E-01 | -1.24E-01 | -7.97E-02 | -4.64E-01 | 9.40E-02 | | 3.62E-01 | 8.41E-01 | 8.72E-01 | 3.11E-01 | 2.70E-02 | 7.83E-01 | 9.25E-01 | 8.11E-01 | 4.90E-01 | 6.60E-01 | 8.52E-01 | 2.80E-01 |
| **LT** | -2.40E-01 | 4.50E-01 | 3.40E-01 | 1.30E-01 | -3.10E-01 | -6.71E-01 | -1.20E-01 | -1.56E-03 | 1.30E-01 | | 5.50E-01 | 2.54E-01 | 1.25E-01 | 4.01E-01 | 2.33E-01 | 1.95E-02 | 1.78E-01 | 2.11E-02 | 2.91E-08 | 3.80E-08 | 2.20E-01 | 2.44E-01 | 5.20E-01 |
| **TD** | -4.30E-01 | 2.60E-01 | -6.90E-02 | -2.30E-01 | -1.50E-01 | -5.00E-01 | -3.52E-01 | -1.99E-01 | | 3 E-01 | 4.30E-03 | 1.96E-01 | 3.53E-02 | 1.71E-01 | 6.85E-03 | 4.93E-01 | 8.15E-01 | 8.75E-01 | 4.41E-02 | 1.10E-02 | 2.40E-04 | 2.64E-01 | 3.70E-02 |
| **LWC** | 7.70E-01 | 1.50E-01 | 8.70E-02 | 3.90E-03 | 3.70E-01 | 3.74E-01 | 8.01E-01 | | 1.20E-01 | 9.90E-01 | 6.10E-01 | 6.29E-01 | 4.86E-03 | 7.69E-14 | 1.88E-07 | 8.08E-01 | 8.66E-02 | 9.27E-01 | 3.22E-01 | 1.20E-01 | 3.20E-39 | 2.18E-01 | 2.20E-07 |
| **LW** | 7.50E-01 | -1.80E-01 | -8.60E-02 | 8.30E-02 | 3.90E-01 | 7.03E-01 | | 1.01E-21 | 2.09E-03 | 2.40E-01 | 3.50E-01 | 6.56E-01 | 9.06E-05 | 3.21E-14 | 1.44E-32 | 4.55E-01 | 3.53E-01 | 5.51E-01 | 2.46E-01 | 4.80E-01 | 3.40E-57 | 1.27E-01 | 2.80E-12 |
| **$A_{max}$** | 6.50E-01 | -7.60E-01 | -8.70E-02 | -5.20E-01 | 6.30E-01 | | 2.51E-06 | 5.45E-02 | 2.10E-02 | 6.20E-04 | 4.10E-01 | 3.01E-01 | 7.12E-04 | 2.59E-02 | 2.96E-03 | 8.87E-01 | 7.09E-02 | 3.18E-01 | 1.70E-04 | 2.20E-04 | 1.40E-05 | 2.00E-01 | 1.10E-03 |
| **SD** | 6 E-01 | -2.50E-01 | -5.10E-01 | -1.10E-01 | | 5.27E-05 | 6.30E-05 | 2.51E-03 | 2.44E-01 | 6.90E-03 | 9.40E-01 | 2.37E-01 | 8.32E-04 | 1.02E-06 | 6.16E-01 | 2.71E-02 | 6.74E-01 | 1.74E-02 | 4.89E-02 | 3.10E-02 | 1.20E-09 | 2.91E-02 | 2.30E-07 |
| **SL** | 1.70E-01 | -1.30E-01 | 6.70E-01 | | 4.70E-01 | 1.95E-01 | 5.88E-01 | 9.87E-01 | 2.56E-01 | 4.30E-01 | 3 E-01 | 3.56E-01 | 3.79E-01 | 1.65E-01 | 3.68E-01 | 5.89E-01 | 3.21E-01 | 6.94E-01 | 1.48E-02 | 9.40E-01 | 3.80E-01 | 6.96E-01 | 10 E-01 |
| **SW** | -1.90E-02 | 8.60E-02 | | 2.60E-05 | 2.70E-03 | 8.50E-01 | 6.39E-01 | 7.32E-01 | 7.48E-01 | 9.40E-02 | 4 E-01 | 6.33E-01 | 1.80E-02 | 3.27E-01 | 9.82E-01 | 5.87E-01 | 8.46E-01 | 1.27E-02 | 8.09E-01 | 6.40E-01 | 6.50E-01 | 4.34E-01 | 3.50E-01 |
| **LWA** | -2.20E-01 | | 7.40E-01 | 5.80E-01 | 4.90E-02 | 4.23E-06 | 9.20E-02 | 1.44E-01 | 4.17E-02 | 1.80E-04 | 2 E-01 | 7.64E-01 | 1.13E-01 | 2.15E-01 | 3.52E-01 | 2.97E-01 | 1.75E-01 | 1.75E-01 | 2.37E-03 | 2.90E-11 | 1.70E-01 | 9.33E-01 | 3.10E-01 |
| **TC** | | 3.40E-02 | 9.20E-01 | 2.90E-01 | 6.90E-11 | 2.00E-05 | 1.54E-34 | 2.23E-19 | 1.77E-04 | 2 E-02 | 1.00E+00 | 9.88E-01 | 4.39E-05 | 1.13E-14 | 2.23E-10 | 1.10E-02 | 2.00E-01 | 5.09E-01 | 2.89E-01 | 7.50E-02 | 8.50E-51 | 3.84E-01 | 9.70E-10 |

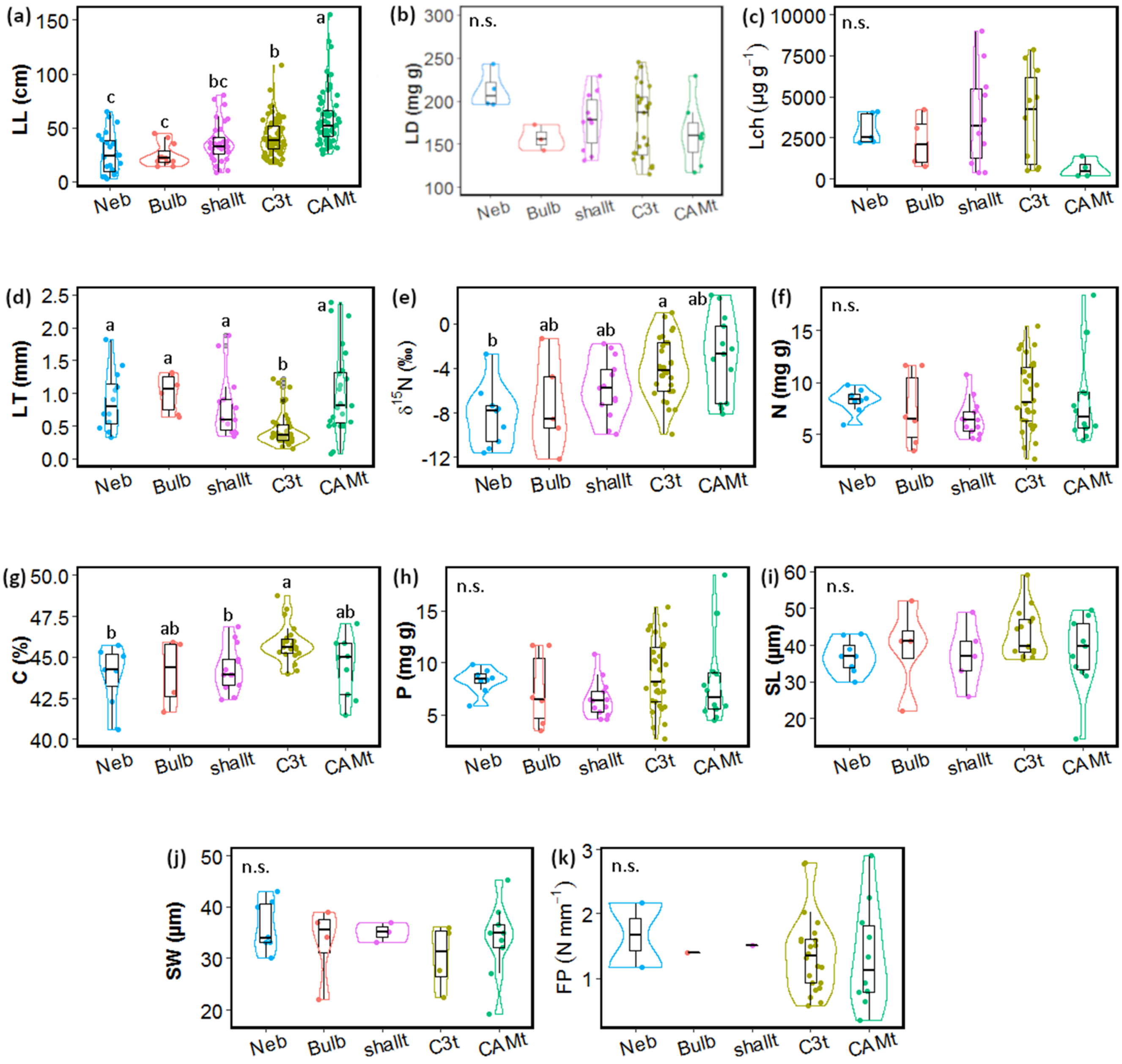


**Figure S1.** Comparison of functional traits between five epiphytic Bromeliaceae functional groups. Neb=nebulophytes; Bulb=pseudobulbs; ShallT=shallow tanks; C3T=$C_3$ tank and CAMT=CAM tanks. FP=force to punch; LD=leaf dry matter content; SLA=specific leaf area; C=leaf carbon content per leaf dry mass; LCh=leaf chlorophyll content per leaf dry mass; LL=leaf length; $\delta^{15}N$=leaf nitrogen isotope signature; N=leaf nitrogen content per leaf dry mass; P=leaf phosphorus content per leaf dry mass; SL=stomatal length; SW=stomatal width. Groups with different letters are significantly different (Wilcoxon post-hoc test, $p < 0.05$), n.s.= non-significant differences.

References used to compile data


1. Adams, W. W.; Martin, C. E. Physiological consequences of changes in life form of the Mexican epiphyte *Tillandsia deppeana* (Bromeliaceae). *Oecologia* **1986**, *70*, 298-304.
2. Agudelo, C. M.; Benavides, A. M.; Taylor, T.; Feeley, K. J.; Duque, A. Functional composition of epiphyte communities in the Colombian Andes. *Ecol.* **2019**, *100*, e02858. 10.1002/ecy.2858
3. Aoyama, E. M.; Sajo, M. Estructura foliar *de Aechmea* Ruiz & Pav. subgênero *Lamprococcus* (Beer) Baker e espécies relacionadas (Bromeliaceae). *Braz. J. Bot.* **2003**, *26*, 461-473.
4. Belmonte, E.; Arriaza, B.; Arismendi, M.; Sepúlveda, G. Foliar Anatomy of Three Native Species of *Tillandsia* L. from the Atacama Desert, Chile. *Plants* **2022**, *11*, 870.
5. Benzing, D. H.; Renfrow, A. The mineral nutrition of Bromeliaceae. *Bot. Gaz.* **1974,** *135*, 281-288.
6. Benzing, D. H.; Friedman, W. E. Patterns of foliar pigmentation in Bromeliaceae and their adaptive significance. *Selbyana* **1981**, *5*, 224-240.
7. Benzing, D. H.; Davidson, E. A. Oligotrophic *Tillandsia circinnata* Schlecht (Bromeliaceae): an assessment of its patterns of mineral allocation and reproduction. *Am. J. Bot.* **1979,** *66*, 386-397.
8. Benzing, D. H. An investigation of two bromeliad myrmecophytes: *Tillandsia butzii* Mez*, T. caput-medusae* E. Morren, and their ants. *Bull. Torrey Bot. Club*. **1970**, 109-115.
9. Benzing, D. H. Bromeliaceae: *Profile of an Adaptive Radiation*. Cambridge University Press, UK, 2000, pp.710
10. Benzing, D. H.; Burt, K. M. Foliar permeability among twenty species of the Bromeliaceae. *Bull. Torrey Bot. Club*. **1970**, 269-279.
11. Braga, M. M. N. Anatomia foliar de Bromeliaceae da Campina. *Acta Amazon.* **1977**, *7*, 5-74.
12. Brighigna, L.; Cecchi Fiordi, A.; Palandri, M. R. Structural characteristics of the mesophyll in some *Tillandsia* species. *Phytomorphology* **1984**, *34*, 191-200.
13. Caballero-Rueda, L. M.; Rodríguez, N.; Martin, C. Dinámica de elementos en epifitos de un bosque altoandino de la Cordillera Oriental de Colombia. *Caldasia* **1997**, *19*, 311-322.
14. Cach-Pérez, M. J.; Andrade, J. L.; Cetzal-Ix, W.; Reyes-García, C. Environmental influence on the inter- and intraspecific variation in the density and morphology of stomata and trichomes of epiphytic bromeliads of the Yucatan Peninsula. *Bot. J. Linn. Soc.* **2016**, *181*, 441-458.
15. Cardelús C.L.; Mack, M. The nutrient status of epiphytes and their host trees along an elevational gradient in Costa Rica. *Plant Ecol.* **2010**, *207*: 25-37.
16. Casañas, O. L.; Jáuregui, D. Morfoanatomía foliar de epífitas presentes en un bosque nublado, Altos de Pipe, Estado Miranda, Venezuela. *Acta Bot. Venez.* **2011**, *34*,153-176.
17. Leroy, C.; Gril, E.; Si Ouali, L.; Coste, S.; Gérard, B; Maillard, P.; Mercier, H.; Stahl, C.; Water and nutrient uptake capacity of leaf-absorbing trichomes vs. roots in epiphytic tank bromeliads. *Environ. Exp. Bot.* **2019**, *163*, 112-123.
18. Cogliatti-Carvalho, L.; Rocha-Pessôa, T. C.; Nunes-Freitas, A. F.; Rocha, C. F. D. Volume de água armazenado no tanque de bromélias, em restingas da costa brasileira. *Acta Bot. Bras.* **2010**, *24*, 84-95.
19. Crayn, D.M.; Winter, K.; Schulte, K.F.; Smith, J.A. Photosynthetic pathways in Bromeliaceae: phylogenetic and ecological significance of CAM and C3 based on carbon isotope ratios for 1893 species. *Bot. J. Linn. Soc.* **2015**, *178*, 169-221.
20. Crayn, D. M.; Winter, K.; Smith, J. A. C. Multiple origins of crassulacean acid metabolism and the epiphytic habit in the Neotropical family Bromeliaceae. *Proc. Natl. Acad. Sci. U.S.A* **2004**, *101*, 3703-3708.
21. de Oliveira, M. L.; de Melo, E. J.; Miguens, F. C. *Tillandsia stricta* Sol (Bromeliaceae) leaves as monitors of airborne particulate matter—A comparative SEM methods evaluation: Unveiling an accurate and odd HP-SEM method. *Microsc. Res. Tech.* **2016**, *79*, 869-879

22. de Oliveira, R. S.; de Oliveira Souza, S.; Aona, L. Y. S.; Souza, F. V. D.; Rossi, M. L.; de Souza, E. H. Leaf structure of *Tillandsia* species (Tillandsioideae: Bromeliaceae) by light microscopy and scanning electron microscopy. *Microsc. Res. Tech.* **2022**, *85*, 253-269.
23. Dézerald, O.; Talaga, S.; Leroy, C.; Carrias, J. F.; Corbara, B.; Dejean, A.; Céréghino, R. Environmental determinants of macroinvertebrate diversity in small water bodies: insights from tank-bromeliads. *Hydrobiologia* **2014**, *723*, 77-86.
24. Díaz-Álvarez, E. A.; de la Barrera, E. Drying protocol does not alter plant δ13C and δ15N: a baseline survey for ecological studies. *Isotopes Environ. Health. Stud.* **2019**, *55*, 526-531.
25. Einzmann, H. J. R.; Beyschlag, J.; Hofhansl, F.; Wanek, W.; Zotz, G. Host tree phenology affects vascular epiphytes at the physiological, demographic and community level. *AoB Plants* **2015**, *7*, plu073, https://doi.org/10.1093/aobpla/plu073
26. Felix, J. D.; Avery, G. B.; Mead, R. N.; Kieber, R. J.; Willey, J. D. Nitrogen content and isotopic composition of Spanish Moss (*Tillandsia usneoides* L.): reactive nitrogen variations and source implications across an urban coastal air shed. *Environ. Process*. **2016**, *3*, 711-722.
27. Gómez, M. A.; Winkler, S. Bromelias en manglares del Pacífico de Guatemala. *Rev. Biol. Trop.* **1991**, 207-214.
28. Griffiths, H. Carbon balance during CAM: an assessment of respiratory $CO_2$ recycling in the epiphytic bromeliads *Aechmea nudicaulis* and *Aechmea fendleri. Plant Cell Environ.* **1988**, *11*, 603-611.
29. Griffiths, H.; Lüttge, U.; Stimmel, K. H.; Crook, C. E.; Griffiths, N. M.; Smith, J. A. C. Comparative ecophysiology of CAM and C3 bromeliads. III. Environmental influences on CO2 assimilation and transpiration. *Plant Cell Environ.* **1986**, *9*, 385-393.
30. Griffiths, H.; Smith, J. A. C.; Lüttge, U.; Popp, M.; Cram, W. J.; Diaz, M. A.; Lee, H. S. L.; Medina, E.; Schäfer, C.; Stimmel, K. H. Ecophysiology of xerophytic and halophytic vegetation of a coastal alluvial plain in northern Venezuela. IV. *Tillandsia flexuosa* Sw. and *Schomburgkia humboldtiana* Reichb., epiphytic CAM plants. *New Phytol.* **1989, *111***, 273-282.
31. Hietz, P.; Wanek, W.; Popp, M. Stable isotopic composition of carbon and nitrogen and nitrogen content in vascular epiphytes along an altitudinal transect. *Plant Cell Environ.* **1999**, *22*, 1435–1443.
32. Hietz P.; Wanek W. Size-dependent variation of carbon and nitrogen isotope abundances in epiphytic bromeliads. *Plant Biol.* **2003**, *5*, 137-142.
33. Husk, G. J.; Weishampel, J. E.; Schlesinger, W. H. Mineral dynamics in Spanish moss, *Tillandsia usneoides* L. (Bromeliaceae), from Central Florida, USA. *Sci. Total Environ.* **2004**, *321*, 165-172.
34. Adams, W. W.; Martin, C. E. Morphological changes accompanying the transition from juvenile (atmospheric) to adult (tank) forms in the Mexican epiphyte *Tillandsia deppeana* (Bromeliaceae). *Am. J. Bot.* **1986**, *73*, 1207-1214.
35. Isley, P. T. *Tillandsia: the world's most unusual air plants*, 1rst ed; Botanical Press, 1987; 256 pp.
36. Latorre, C.; González, A. L.; Quade, J.; Fariña, J. M.; Pinto, R.; Marquet, P. A. Establishment and formation of fog-dependent *Tillandsia landbeckii* dunes in the Atacama Desert: Evidence from radiocarbon and stable isotopes. *J. Geophys. Res. Biogeosciences* **2011**,*116*, 1-12, doi:10.1029/2010JG001521
37. Leroy, C.; Carrias, J. F.; Céréghino, R.; Corbara, B. The contribution of microorganisms and metazoans to mineral nutrition in bromeliads. *J. Plant Ecol.* **2016**, *9*, 241-255.
38. Leroy, C.; Carrias, J. F.; Corbara, B.; Pélozuelo, L.; Dézerald, O.; Brouard, O.; Céréghino, R. Mutualistic ants contribute to tank-bromeliad nutrition. *Ann, Bot.* **2013**, *112*, 919-926.
39. Leroy, C.; Corbara, B.; Dejean, A.; Céréghino, R. Ants mediate foliar structure and nitrogen acquisition in a tank-bromeliad. *New Phytol.* **2009**, *183,* 1124-1133.
40. López-Ferrari, A. R.; Espejo-Serna, A.; Blanco, P. Circunscripción de *Tillandsia chaetophylla* Mez y descripción de *Tillandsia sessemocinoi* (Bromeliaceae: Tillandsioideae). *Acta Bot. Mex.* **2006**, *76*, 77-88.
41. Males, J.; Griffiths, H. Economic and hydraulic divergences underpin ecological differentiation in the Bromeliaceae. *Plant Cell Environ.* **2018**, *41*, 64-78.

42. Males J. Structure-function relationships in the water-use strategies and ecological diversity of the Bromeliaceae. PhD thesis, University of Cambridge. Cambridge, UK. 2017.
43. Marino, N. A.; Guariento, R. D.; Dib, V.; Azevedo, F. D.; Farjalla, V. F. Habitat size determine algae biomass in tank-bromeliads. *Hydrobiologia* **2011**, *678*, 191-199.
44. Martin, C. E. Physiological Ecology of the Bromeliaceae. *Bot. Rev.* **1994**, *60*, 1-82.
45. Martins, J. P. R.; Martins, A. D.; Pires, M. F.; Braga Junior, R. A.; Reis, R. O.; Dias, G. D. M. G.; Pasqual, M. Anatomical and physiological responses of *Billbergia zebrina* (Bromeliaceae) to copper excess in a controlled microenvironment. *Plant Cell, Tissue Organ Cult.* **2016**, *126*, 43-57.
46. Matos, J.A.; Rudolph, D.C. *Aspects of the life history of Tillandsia deppeana. In* Gardner, C.S. (Ed.), World Bromeliad Conference. Mission Press, Corpus Christi, TX, USA, 1984, pp. 71–75.
47. Maxwell, K.; Griffiths, H.; Borland, A. M.; Young, A. J.; Broadmeadow, M. S. J.; Fordham, M. C. Short-term photosynthetic responses of the C3-CAM epiphyte *Guzmania monostachia* var. *monostachia* to tropical seasonal transitions under field conditions. *Aust. J. Plant Physiol.* **1995**, *22*, 771-781.
48. Meisner, K. Functional relevance of heteroblasty in Bromeliaceae. PhD thesis, Carl von Ossietzky Universität, Oldenburg. 2012.
49. Nuzhyna, N. V.; Kolomiyets, T. V. Differences in anatomical and morphological structure of plants from the genus Neoregelia LB Sm. (Bromeliaceae) as a result of adaptation to arid environmental conditions. *Mod. Phytomorphol.* **2015**, *8*, 137-144.
50. Palací, C. A.; Brown, G. K.; Tuthill, D. E. Vegetative Morphology and Leaf Anatomy of *Catopsis* (Tillandsioideae: Bromeliaceae). *Selbyana* **2004**, *25*,138–50.
51. Pereira-Zaldívar, N. A.; Patiño-López, L. D.; Rodríguez-García, R.; Andrade, J. L.; Cach-Pérez, M. J.; Espadas-Manrique, C.; Barredo-Pool, F.; Reyes-García, C. Life form and anatomical traits related to trichome mediated external water transport in epiphytic Bromeliaceae. *Plant, cell and environ.* 2022 (Submitted).
52. Pereira, T. A. R.; de Oliveira, T. S.; da Silva, L. C.; Azevedo, A. A. Comparative leaf anatomy of four species of Bromelioideae (Bromeliaceae) occurring in the Atlantic Forest. *Brazil. Bot.* 2011, *89,* 243-253.
53. Petter, G.; Wagner, K.; Wanek, W.; Sánchez Delgado, E. J.; Zotz, G.; Cabral, J. S.; Kreft, H. Functional leaf traits of vascular epiphytes: Vertical trends within the forest, intra- and interspecific trait variability, and taxonomic signals. *Func. Ecol.* **2016**, *30,* 188-198.
54. Pierce, S. The jeweled armor of Tillandsia—Multifaceted or elongated trichomes provide photoprotection. *Aliso: A J. Syst. Floristic Bot.* **2007**, *23*, 44-52.
55. Pierce, S.; Winter, K.; Griffiths, H. Carbon isotope ratio and the extent of daily CAM use by Bromeliaceae. *New Phytol.* **2002**, 156, 75-83.
56. Proença, S. L.; Sajo, M. D. G. Anatomia foliar de bromélias ocorrentes em áreas de cerrado do Estado de São Paulo, Brasil. *Acta Bot. Brasil.* **2007**, *21*, 657-673.
57. Puccio, P. *Tillandsia tenuifolia* L. Monaco nature Encyclopedia. Available online: https://www.monaconatureencyclopedia.com/tillandsia-tenuifolia/?lang=es (accessed on July 2022).
58. Rammler H. Ökophysiologie von Epiphyten und Bäumen in einem Bergregenwald in Mexiko und einem Tieflandregenwald in Costa Rica. Master thesis, Universität für Bodenkultur, Wien. 2004.
59. Reyes-García, C. Ecofisiología de epifitas de selva baja caducifolia del género *Tillandsia* (bromeliaceae): Estacionalidad y fotosíntesis. Bachelor's thesis. Universidad nacional Autónoma de México, México. 2001.
60. Ribeiro, F.; Aoyama, E. Anatomia foliar de Bromeliaceae da trilha do rio Timbuí na Estação Biológica de Santa Lúcia, Santa Teresa–es. *Enciclopédia Biosfera* **2019**, *16*, 185-197.
61. Rosado-Calderón, A. T.; Tamayo-Chim, M.; de la Barrera, E.; Ramírez-Morillo, I. M.; Andrade, J. L.; Briones, O.; Reyes-García, C. High resilience to extreme climatic changes in the CAM epiphyte *Tillandsia utriculata* L. (Bromeliaceae). *Physiol. Plant.* **2020**, *168*, 547-562.
62. Schmidt, G.; Stuntz, S.; Zotz, G. Plant size - an ignored parameter in epiphyte ecophysiology. *Plant. Ecol.* **2001**, 153, *65*-72.

63. Silva, E. C. *Aechmea bromeliifolia* (Rudge) Baker (Bromeliaceae) cultivada in vitro e ex vitro: morfologia, anatomia e ultraestrutura. Master thesis. Universidade Federal de Goiás, Goiânia, Brazil, 2016.
64. Smith, J. A. C.; Griffiths, H.; Lüttge, U.; Crook, C. E.; Griffiths, N. M.; Stimmel, K. H. Comparative ecophysiology of CAM and C3 bromeliads. IV. Plant water relations. *Plant Cell Environ.* **1986**, *9*, 395-410.
65. Souza, G. M. D.; Estelita, M. E. M.; Wanderley, M. D. G. L. Anatomia foliar de espécies brasileiras de *Aechmea* subg. Chevaliera (Gaudich. ex Beer) Baker, Bromelioideae-Bromeliaceae. *Braz. J. Bot.* **2005**, *28*, 603-613.
66. Stefano, M.; Papini, A.; Brighigna, L. A new quantitative classification of ecological types in the bromeliad genus Tillandsia (Bromeliaceae) based on trichomes. *Rev. Biol. Trop.* **2008**, *56*, 191-203.
67. Stuntz, S.; Zotz, G. Photosynthesis in vascular epiphytes - a survey of 27 species of diverse taxonomic origin. *Flora* **2001**, *196*, 132-141.
68. Susan-Tepetlan, T. M.; Velázquez-Rosas, N.; Krömer, T. Cambios en las características funcionales de epífitas vasculares de bosque mesófilo de montaña y vegetación secundaria en la región central de Veracruz, México. *Bot. Sci.* **2015**, *93*, 1-11.
69. Terreros-Olivares, L. Revisión sistemática del complejo de especies de *Tillandsia juncea* (Ruiz & Pav.) Poir. Bromeliaceae. Master thesis, Universidad Metropolitana de México, México, 2012.
70. Versieux, L. M.; Medeiros, A. S. M. Leaf anatomical characterization of *Guzmania* Ruiz & Pav. and *Mezobromelia* LB Sm. (Tillandsioideae, Bromeliaceae). *J. Bromeliad Soc.* **2017**, *67*, 8-26
71. Vite-Posadas, J. A.; Brechú-Franco, A. E.; Laguna-Hernández, G.; Rojas-Bribiesca, M. G.; Osuna-Fernández, H. R. Morphoanatomical characterization and antimicrobial activity of *Tillandsia imperialis* (Bromeliaceae). *Polibotánica* **2011**, *31*, 20-29.
72. Wanek, W.; Zotz, G. Are vascular epiphytes nitrogen or phosphorus limited? A study of plant 15N fractionation and foliar N: P stoichiometry with the tank bromeliad Vriesea sanguinolenta. *New Phytol.* **2011**, *192*, 462-470.
73. Wania, R.; Hietz, P.; Wanek, W. Natural 15N abundance of epiphytes depends on the position within the forest canopy: source signals and isotope fractionation. *Plant Cell Environ.* **2002**, *25*, 581-589.
74. Wester, S.; Mendieta-Leiva, G.; Nauheimer, L.; Wanek, W.; Kreft, H.; Zotz, G. Physiological diversity and biogeography of vascular epiphytes at Río Changuinola. *Panama Flora* **2011**, *206*, 66–79.
75. Zambrano, A. R. C.; Linis, V. C.; Nepacina, M. R. J.; Silvestre, M. L. T.; Foronda, J. R. F.; Janairo, J. I. B. Wetting properties and foliar water uptake of *Tillandsia* L. *Biotribology* **2019**, *19*, 100103.
76. Zorger, B. B.; Arrivabene, H. P.; Milanez, C. R. D. Adaptive morphoanatomy and ecophysiology of *Billbergia euphemiae*, a hemiepiphyte Bromeliaceae. *Rodriguésia* **2019**, *70*, 1-10, doi.org/10.1590/2175-7860201970091.
77. Zotz G. How prevalent is crassulacean acid metabolism among vascular epiphytes? *Oecologia* **2004**, *138*, 184-192.
78. Zotz, G.; Asshoff, R. Growth in epiphytic bromeliads: response to the relative supply of phosphorus and nitrogen. *Plant Biol.* **2010**, *12*, 108-113.
79. Zotz, G.; Ziegler, H. The occurrence of crassulacean acid metabolism among vascular epiphytes from Central Panama. *New Phytol.* **1997**, *137*, 223-229.
80. Zotz, G.; Enslin, A.; Hartung, W.; Ziegler, H. Physiological and anatomical changes during the early ontogeny of the heteroblastic bromeliad, *Vriesea sanguinolenta*, do not concur with the morphological change from atmospheric to tank form. *Plant Cell Environ.* **2004**, *27*, 1341-1350.
81. Zotz, G.; Leja, M.; Aguilar-Cruz, Y.; Einzmann, H. J. How much water is in the tank? An allometric analysis with 205 bromeliad species. *Flora* **2020**, *264,* 151557, https://doi.org/10.1016/j.flora.2020.151557
